# Genome diversity of marine phages recovered from Mediterranean metagenomes: Size matters

**DOI:** 10.1371/journal.pgen.1007018

**Published:** 2017-09-25

**Authors:** Mario López-Pérez, Jose M. Haro-Moreno, Rafael Gonzalez-Serrano, Marcos Parras-Moltó, Francisco Rodriguez-Valera

**Affiliations:** 1 Evolutionary Genomics Group, División de Microbiología, Universidad Miguel Hernández, Campus de San Juan, San Juan de Alicante, Spain; 2 Centro de Biología Molecular 'Severo Ochoa' (Consejo Superior de Investigaciones Científicas and Universidad Autónoma de Madrid), Cantoblanco, Madrid, Spain; Technion, ISRAEL

## Abstract

Marine viruses play a critical role not only in the global geochemical cycles but also in the biology and evolution of their hosts. Despite their importance, viral diversity remains underexplored mostly due to sampling and cultivation challenges. Direct sequencing approaches such as viromics has provided new insights into the marine viral world. As a complementary approach, we analysed 24 microbial metagenomes (>0.2 μm size range) obtained from six sites in the Mediterranean Sea that vary by depth, season and filter used to retrieve the fraction. Filter-size comparison showed a significant number of viral sequences that were retained on the larger-pore filters and were different from those found in the viral fraction from the same sample, indicating that some important viral information is missing using only assembly from viromes. Besides, we were able to describe 1,323 viral genomic fragments that were more than 10Kb in length, of which 36 represented complete viral genomes including some of them retrieved from a cross-assembly from different metagenomes. Host prediction based on sequence methods revealed new phage groups belonging to marine prokaryotes like SAR11, Cyanobacteria or SAR116. We also identified the first complete virophage from deep seawater and a new endemic clade of the recently discovered Marine group II Euryarchaeota virus. Furthermore, analysis of viral distribution using metagenomes and viromes indicated that most of the new phages were found exclusively in the Mediterranean Sea and some of them, mostly the ones recovered from deep metagenomes, do not recruit in any database probably indicating higher variability and endemicity in Mediterranean bathypelagic waters. Together these data provide the first detailed picture of genomic diversity, spatial and depth variations of viral communities within the Mediterranean Sea using metagenome assembly.

## Author summary

These data provided a glimpse of the genetic diversity and variability of viral sequences without introducing the amplification biases produced when studying viromes. Metagenomes contain abundant viral material due to cells retrieved while undergoing viral lysis or as temperate viruses inserted in the chromosome. Using very stringent criteria, we have managed to assemble large viral genomes from Mediterranean metagenomes. This large-scale study using direct assembly from metagenomes, i.e. from the cellular fraction, clearly shows that metagenomes are an important tool to study environmental viral communities containing complementary information, which is missing in viromes, that should be taken into consideration when studying viral genetic diversity. Thus, in this study we have described more than 1,300 viral genomic fragments larger than 10Kb, of which 36 represented complete viral genomes, including the first deep sea virophage, one novel Marine Group II euryarchaeota virus (magrovirus) and others infecting Cyanobacteria, SAR11 or SAR116. All these new viral contigs were obtained from a collection of 24 metagenomes representing a broad range of geographical and ecological biomes from the Mediterranean Sea.

## Introduction

Bacteriophages (viruses that infect bacteria), often referred to as phages, are considered the most abundant and diverse biological entities in aquatic systems [1] with an estimated population density of 10^7^ per ml of seawater [2]. They are not only abundant but also important players in the energy and nutrient cycles [1,3–6] through the lysis of host microbial cells, phenomenon designated as ‘‘viral shunt” [7]. Phages also play a critical role in the evolution of bacteria, facilitating horizontal gene transfer and helping to increase genetic diversity in the microbial community [8,9]. Despite their importance, phage genetic diversity, evolution and distribution remains poorly characterized because phages do not share a universal marker gene analogous to the 16S rRNA gene in bacteria and archaea and most of marine microbes are still unculturable under laboratory conditions and therefore also their viruses [10].

Advances in next-generation sequencing have allowed developing culture-free approaches, such as metagenomics, providing a powerful tool that has revolutionized the analysis of microbial communities in several natural environments [11–14]. Large-scale metagenomic studies of marine viruses from both surface [15] and deep ocean [16,17] have advanced in the structure of viral communities, which appears to be more diverse than previously appreciated. Despite those major advances, since the amount of viral DNA recovered is small, viral metagenomes (or viromes) normally need a previous step for DNA amplification, using mostly multiple displacement amplification (MDA), that is likely to produce highly biased samples [18]. Alternative library preparation techniques have been recently developed [10,19]. Although these techniques require ultra-low DNA quantities and introduce only minimal biases, they also have other drawbacks [19,20]. An alternative to all these methodologies is using the viral DNA present in metagenomes in relatively large amounts. It has been previously reported a high presence of viral DNA (around 10% to 15%) in marine metagenomes [21,22] likely belonging to cells that are undergoing lytic cycle [23]. Metagenomes (the fraction > 0.2 μm) will also include (i) viruses using the lysogenic cycle (either integrated or as a plasmid), (ii) viruses attached/adsorbed to particles, and (iii) viruses larger than 0.2 μm. However, the vast majority is probably the replication intermediate generated during the lytic cycle. This natural amplification method increases the amount of viral DNA available that can be cloned into fosmids or assembled. Using this strategy, 206 complete marine phage genomes were recovered from a metagenomic sample from the Mediterranean deep chlorophyll maximum [24] and twenty-eight from two deep (1,000-m and 3,000-m) Mediterranean Sea metagenomic libraries [25]. More recently, a complete set of genomes of a novel group of viruses, designed as magrovirus, that seem to infect the uncultured marine group II Euryarchaeota were retrieved from a cross-assembly of microbial, viral, and transcriptomic datasets [26]. It seems likely that metagenomes contain some important viral information, which is missing in viromes.

In addition, another valuable tool that has emerged in the last decade is single cell genomics. Although still expensive and unreliable, due to the amplification steps, provides the sequences of individual microbes and, if they happen to be infected at the time of sorting, phages as well. This allows linking the phage to the host [27]. For example, a total of 69 SUP05-associated viruses representing five new genera within Caudovirales and Microviridae families were identified using single-cell amplified genomes [28]. Furthermore, a new technique, “viral tagging”, for sorting cells infected by all the phages in a sample has been described improving the analyses of virus-host interactions [29]. This method has been applied for a single strain of *Synechococcus* sp. WH7803 against Pacific Ocean cyanophages, showing an unprecedented viral diversity with at least 26 dsDNA viral populations capable of infecting Cyanobacteria [29]. There is also other recent technological advances for understanding dynamics of phage–host interactions such as phageFISH or microfluidic digital PCR (reviewed in [10]). Other quantitative methods are now available to evaluate viral numbers in a sample e.g. estimate ssDNA virus abundance [30].

The Mediterranean Sea is seasonally oligotrophic and characterized by deep convective winter mixing and summer stratification of the water column. It is also relatively warm and deep, maintaining a relatively high temperature (>13°C) throughout the water column [31]. We previously analysed the deep chlorophyll maximum (DCM) in a single sample taken during fall (October) 2007 [21] by high throughput metagenomics. From the same station in the Mediterranean Sea at different depths of the water column including the DCM we have performed different sampling campaigns (winter and summer) during four consecutive years (from 2012–2015). Several new groups of microbes have been later described using assembly of Illumina high coverage metagenomes and metagenomic fosmid clones [32–35]. Recently, we took and sequenced samples from a depth profile every 15 meters, including also two additional samples from 1,000 and 2,000 meters at a single site in the off-shore Western Mediterranean. By high-throughput metagenomics, we were able to study the structure of the community, evaluate the presence of some ecologically relevant genes and reconstruct the genomes of representative microbes [36]. Although the purpose of these studies was only the description of the bacterial populations, we have found a considerable proportion of assembled contigs related to viruses in all the metagenomes. Thus, in this study we have described more than 1,300 viral genomic fragments larger than 10Kb, of which 36 represented complete viral genomes. Besides, we also included in the analysis five more samples from both, DCM and deep (aphotic zone) waters, collected along the Eastern Mediterranean Sea. These data provided a glimpse of the genetic diversity and variability of these putative phage sequences without any previous amplification step. This large-scale study using direct assembly from metagenomes i.e. from the cellular fraction, clearly shows that metagenomes are an important tool to study environmental viral communities containing complementary information which is missing in viromes that should be taken into consideration when studying viral genetic diversity in order to better understand the ecological roles played by viruses in the environment.

## Results and discussion

We have compared a collection of 24 metagenomes representing a broad range of geographical and ecological biomes from the Mediterranean Sea. The samples analysed were taken from six sampling sites from the Eastern and Western Mediterranean and collected from different depths (15 to 3,500 m), filter pore size, and season (stratified or mixed). Metadata of the samples are summarized in S1A and S1B Fig. Metagenomes were classified by depth into: upper photic (UP), deep chlorophyll maximum (DCM), lower photic (LP), meso- and bathypelagic waters (DEEP) and MIX when the water column is mixed, i.e. in winter when the water column is not thermally stratified. We have included also two viromes to compare. These viromes were obtained from DCM depths at the same site in 2011 (MedDCM-Vir-MDA) [24] using MDA and another in 2013 without any treatment (MedDCM-SEP2013-Vir).

**Fig 1:**
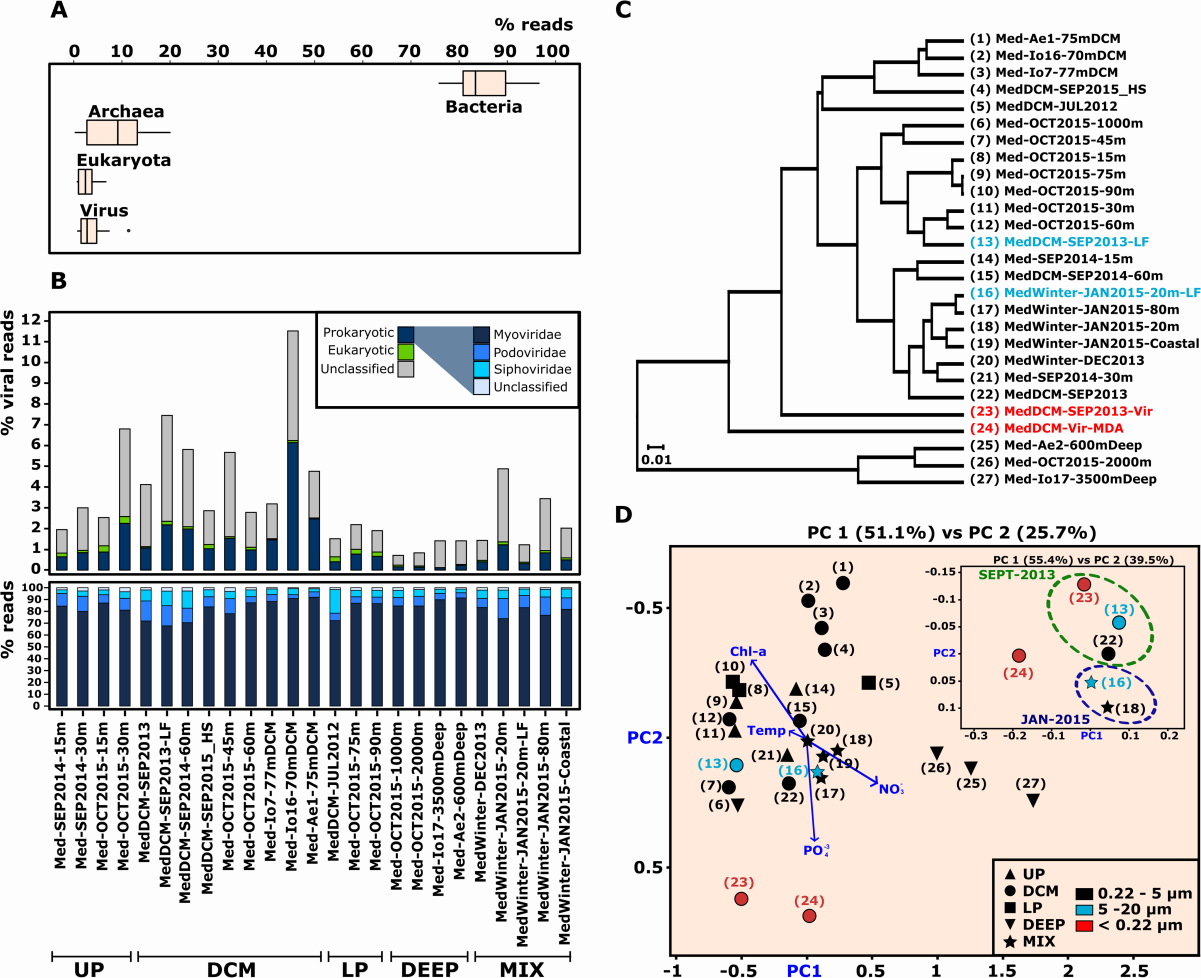
Taxonomic analyses based on metagenomic reads. Taxonomic affiliation was obtained matching the reads against the NR database (>50% identity, >50% alignment). (**A**) Bacterial, archaeal, eukaryal and viral box plots were done using the 24 metagenomic data sets described in S1 Fig. (**B**) Upper panel shows the percentage of viral reads classified for each sample. Relative abundance of prokaryotic viral families is showed in more detail in the bottom panel. Using only reads assigned as a viral origin (**C**) UPGMA taxonomic tree and (**D**) PCoA were inferred with the cluster analysis option in MEGAN6 and a Bray-Curtis ecological distance matrix. Samples highlighted in blue and red correspond to samples obtained from the 5–20 μm and <0.22 μm filters, respectively. UP: Upper Photic, DCM: Deep Chlorophyll Maximum, LP: Lower Photic, DEEP: deep samples, MIX: mixed samples (in winter, without stratification).

As expected, the percentage of total metagenomic reads that could be annotated and attributed to the prokaryotic fraction (bacteria and archaea) in all these metagenomes was more than 80%. Eukaryotic and viral sequences accounted for <10% of the reads while Archaea reached up to 20% in some samples (Fig 1A). The DCM, the zone of maximal phytoplankton concentration [37], was the region with greater abundance of viral reads (from 2.8 to 11.7%), while in deeper waters (LP and DEEP) their presence was significantly smaller (Fig 1B). Independently of the depth, filter pore size or water column region, we observed a dominance of dsDNA viruses of the order Caudovirales, mostly Myoviridae, which accounted for 67%–92% of the viral reads detected (Fig 1B).

Comparison based on sequence similarity using only those reads derived from viral origin (Fig 1C and 1D) revealed four well-differentiated clusters. DCM samples from the Eastern Mediterranean Sea (Samples 1, 2 and 3) share high level of similarity among them, probably due to the ultraoligotrophic conditions of the easternmost part as seen in Fig 1D in which the Eastern Mediterranean samples are located opposite to the abundance of inorganic nutrients (phosphate and nitrate). Winter MIX samples grouped together as well, correlated with NO_3_^-^ concentration. The most distinct metagenomic samples were the viromes and deep metagenome samples (Fig 1C and 1D). Chl-a was the main environmental factor that influenced both, DCM and UP samples, as could be expected.

### Comparison of viral metagenomic reads obtained from three different filter fractions

Frequently, in marine samples, seawater is sequentially filtered using different pore-sizes to separate different size fractions. For example, planktonic macroorganims (>20.0 μm), eukaryotic cells and particle-associated microbes (5.0–20.0 μm), free-living prokaryotic communities (0.22–5.0 μm) and finally the viral pool is concentrated by ultrafiltration. The comparison of the size-fractionated microbial communities in marine metagenomes showed that viral DNA was overrepresented in the particle-associated fraction [22,38]. This phenomenon has been attributed to the presence of more eukaryotic DNA and can be interpreted as a reflection of higher infection rate in this cellular fraction [38–41]. Using only metagenomic reads considered of viral origin obtained from the three different filter fractions (5.0–20.0 μm, 0.22–5.0 μm and <0.22 μm) from the same seawater sample collected in September 2013 from the DCM in the western Mediterranean Sea, we could analyse the viral diversity across size fractions (S1A and S1B Fig). The dendrogram (Fig 1C) and the principal coordinate composition (PcoA) (Fig 1D, inset) showed a clear separation between the three filter sizes at the level of the percentage identity of individual reads among metagenomes obtained from the same place and year (Samples 13, 22 and 23). Although the predominant group of viruses in all the samples were attributed to members of the Caudovirales (dsDNA viruses), the two metagenomic samples with larger pore size (MedDCM-SEP2013-LF (Sample 13) and MedDCM-SEP2013 (Sample22)) showed an increase in the percentage of the Myoviridae and a relative decrease in the number of Podoviridae in comparison with the virome (MedDCM-SEP2013-Vir (Sample 23)). As a reference, we have included other virome (MedDCM-Vir-MDA (Sample 24)), obtained from the same place in 2011. However, unlike the other, this sample was amplified by MDA and clearly was enriched in ssDNA viruses (mainly Microviridae), not surprising since MDA samples are known to be highly biased towards the amplification of this kind of viruses [20,42]. Same results were obtained from a second group of samples (16 and 18) collected from the same place at 20m in January (2015), during the mixing of the water column [38]. Although this sample does not have a virome, particle-associated (Sample 16) and free-living (Sample 13) viral community were different between them and also with the summer samples (Fig 1C and 1D, inset). These results show that metagenomes contain some important viral information, which is missing in the viromes and should be taken into consideration if we want to study the complete viral genetic diversity. As previously described and shown by recruitment [26] cellular metagenomes are an excellent source of viral DNA information. It should be noted that these classifications based on reads have some limitations for example the relatively few validated viral sequences deposited in public databases, and provide only a rough estimation of the community. However, these data suggest that different filter sizes contain different viral sequences.

### Assembly of metagenomic viral contigs

Metagenomes were assembled individually resulting in 45,698 contigs larger than 10Kb (S1A Fig). Only 6.7% (3,009) were assigned as putative viral contigs based on similarity to viral sequences deposited in the NCBI nr database. However, in order to avoid chimeric assembly and support the viral origin, we selected only the contigs that (i) contained several hallmark viral genes (i.e terminases, portal protein, tail protein and major capsid proteins) or (ii) syntenic contigs with cultured viral genomes or metagenomic fosmids obtained previously from the Mediterranean Sea [24,25]. Finally, we manually selected 1,323 metagenomic viral contigs for further analysis, ranging from 10 to 196Kb (average contig size 23Kb; GC% range 18–55). It is remarkable that we have found several contigs with high similarity (id > 99%) to uvMED and uvDEEP genomes [24,25] in spite of the time elapsed between sample retrieval (S2 Fig).

A total of 39,949 open reading frames were identified and clustered based on sequence similarity into 20,951 protein clusters, 48% (9,968) of which showed significant homology to sequences present in the pVOGs (Prokaryotic Virus Orthologous Groups) database [16], clearly being virus-related (S1 Table). The highest percentage was classified within the *Myoviridae* family (order Caudovirales) including structural proteins (tail tube protein, baseplate tail tube cap, baseplate wedge subunit) DNA metabolism (DNA endonuclease, helicase) as well as genes involved in nitrogen metabolism during infection in cyanophages (phytanoyl-CoA-dioxygenase and 2OG-Fe(II) oxygenase) [43]. However, the comparison against several other datasets of uncultivated viral genomes [24,25,44,45], including the viral RefSeq, showed that *ca*. 30% of the protein clusters (6,198 of 20,951) were exclusive in our dataset and most of them derived from easternmost and deep metagenomes suggesting a great diversity that remains to be discovered in bathypelagic regions.

### Putative host prediction

**Fig 2:**
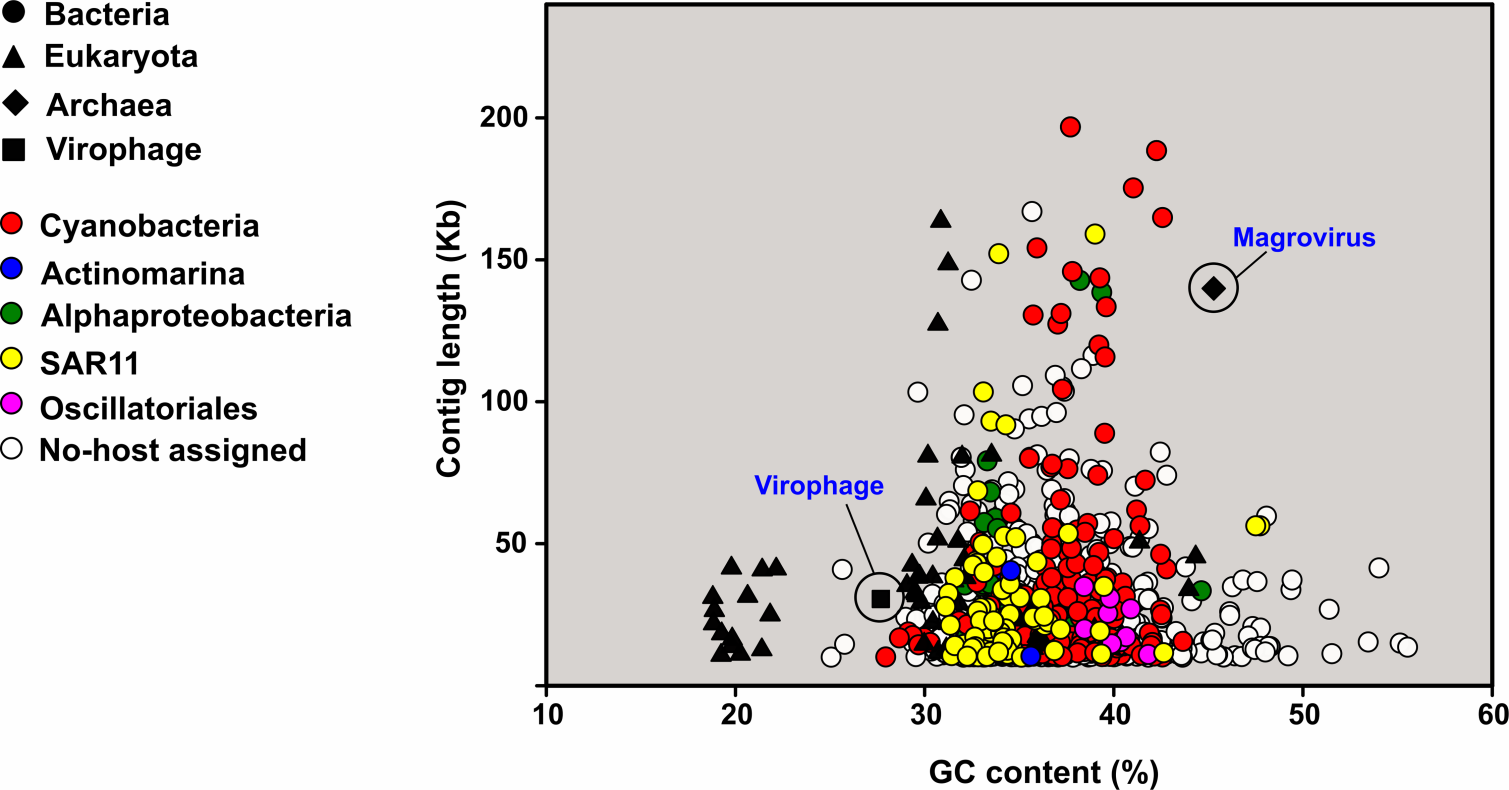
Distribution of metagenomic viral contigs by length (Kb) and GC content (%). Bacterial phages are open circles and colored according to the host assigned.

We constructed a phylogenetic tree using the large-subunit terminase extracted from the contigs (280) in order to evaluate their diversity (S3 Fig), since it has been reported that this gene can be used as a marker to reconstruct phylogeny in tailed bacteriophages [46]. Besides, we included 1,220 sequences belonging to the previous metagenomic fosmid libraries from the Mediterranean Sea [24,25] and some other references (S3 Fig). Most of the terminases contained a Pfam Terminase 6 domain (PF03237) and the closest relatives of the *ca*. 60% of the sequences were terminases from cyanophages. Using a combination of different approaches (see Methods) we were able to assign putative hosts to 438 contigs (*ca*. 33% of the total) (S2 Table and Fig 2). The most frequent host prediction (*ca*. 53%) were Cyanobacteria, followed by Alphaproteobacteria, mainly SAR11. While cyanophages were recovered mostly from the photic zone, we have obtained some pelagiphages also from bathypelagic waters. Twelve sequences could be assigned to SAR116 and three sequences clustered together with HMO-2011, one of the most abundant phages in the ocean [47]. This is not surprising since most of the metagenomes come from the UP and DCM. However, we detected some new and uncharacterized contigs (mostly from deep metagenomic sequences), probably belonging to new lineages. Bathypelagic regions are one of the least understood ecosystems on Earth. They are extreme environments highly oligotrophic and is already known that viral abundance decreases in the deeper water column [1,48]. It is important to emphasize here the peculiar nature of Mediterranean bathypelagic waters due to their relatively warm temperature [31]. The Pacific Ocean virome (POV) dataset that includes samples from the deep Pacific (1,000 to 4,300 m in depth) [16] and more recently a larger dataset including many globally distributed deep-sea viral metagenomes from the Malaspina expedition [45] have provided new insights into viruses from the bathypelagic regions. However, none of the deep samples comes from the Mediterranean Sea. In addition, we found 50 contigs related to eukaryotic viruses with the lowest GC content (S2 Table and Fig 2). These contigs were mainly related to viruses of the Phycodnaviridae family such as *Aureococcus anophagefferens*, *Phaeocystis globosa* and *Micromonas pusilla* virus. Probably these metagenomic viral contigs come from sporadic blooms of marine phytoplankton since they have only been found in a specific metagenome. For example, *Phaeocystis globosa* contigs were only found in the Med-OCT2015-15m metagenome. We also found a new virophage and a virus that putatively infects marine group II archaea (see below).

### Complete genomes and Metagenome-Assembled Viral Genomes (MAVGs)

Due to the fact that we have used several metagenomes to extract the contigs, we first grouped all the sequences into clusters in order to avoid genome redundancy. An all-versus-all comparison was performed using different percentages of identity and we decided to use a criterion of >20% coverage but with a nucleotide sequence identity >90% since the percentage of contigs clustering was similar at 90 and 95% (current cut-off use for a viral population). However, this percentage decreased below 90%. Sequence similarity of the 1,323 contigs resulted in 927 different viral clusters (VCs); 177 with two or more representatives and 751 singletons (S2 Table).

**Fig 3:**
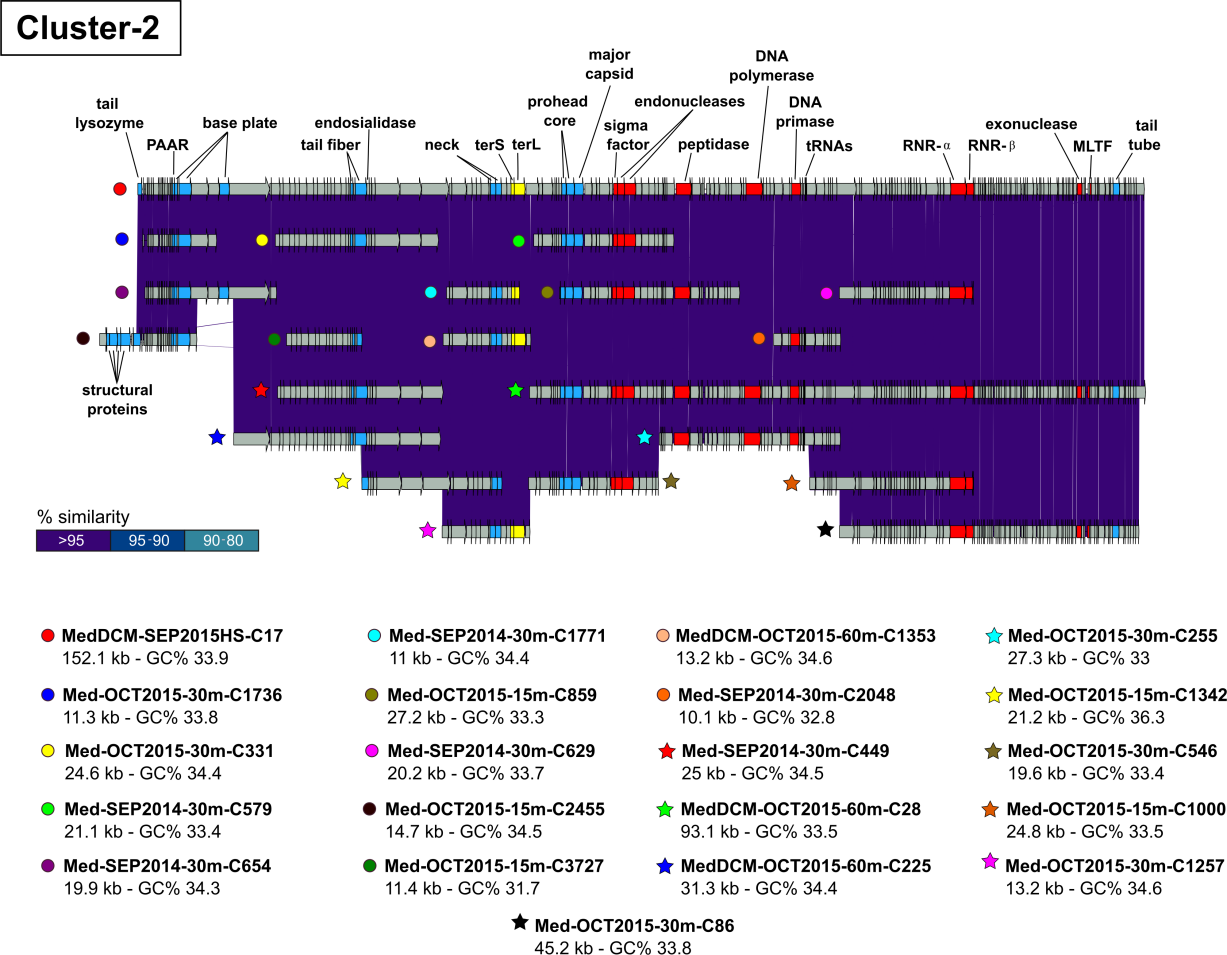
Reconstruction of Metagenome-Assembled Viral Genomes (MAVGs) using sequences belonging to Cluster-2. A nucleotide comparison of several highly related contigs coming from different metagenomic samples is shown. Selected genes are labeled and colored uniformly.

We used two different methods to identify complete genomes from VCs (i) contigs with identical repeated sequences (>30 nt) at the 5′ and 3′ terminal regions that we called complete genome representatives (CGRs following previous nomenclature [24]) and (ii) contigs presenting relative gene order and content similar to database phage genomes that indicate completeness (designated genomic fragments GFs [24]) (Table 1). Moreover, after close manual inspection, we were able to extend the length of some genomes since the identity among contigs was higher than 99%, although they came from different years and depths. Some of these were classified as complete viral genomes by the presence of overlapping terminal regions. Since these complete genomes were retrieved from a cross-assembly of viral contigs belonging to different metagenomes, we called them Metagenome-Assembled Viral Genomes (MAVGs). Five MAVGs coming from clusters 2, 3, 4, 5 and 18 were obtained (Table 1). Fig 3 shows the reconstruction of MAVG-2 using sequences belonging to Cluster-2 coming from different metagenomic samples. This MAVG has been putatively classified as a new pelagiphage similar to HTVC008M (see below). These MAVGs showed large overlaps of nearly identical contigs (>99%) from different samples suggesting that some phage populations can survive during several years showing a remarkable genetic stability. A recent study by [49] using only cyanophage isolates collected from the same locations over a decade revealed similar results showing cyanophage genomic clusters that remained genetically invariant. However, we have shown here that this hypothesis can be extrapolated to other groups such as pelagiphages.

**Table 1:** General features of complete genomes and putative host assignment.

Contig name	Length (bp)	Contig type	GC (%)	#CDSs	tRNA	Putative host
MedDCM-OCT2015-60m-C4	196,757	^a^CGR	37.7	223	6	Cyanobacteria
MedDCM-SEP2014-C2	196,733	CGR	37.7	223	6	Cyanobacteria
Med-SEP2014-30m-C1	188,425	CGR	42.3	216	2	Cyanobacteria
MedWinter-DEC2013-20m-C17	188,424	CGR	42.3	216	2	Cyanobacteria
Med-OCT2015-15m-C9	175,282	CGR	41.0	214	6	Cyanobacteria
Med-Io7-70mDCM-C14	159,013	CGR	39.0	160	5	SAR11
Med-OCT2015-15m-C16	142,663	CGR	38.2	197	2	Alphaproteobacteria
Med-OCT2015-90m-C1	139,890	CGR	45.3	172	2	Marine group II Euryarchaeota
Med-OCT2015-15m-C18	138,459	CGR	39.4	176	2	Alphaproteobacteria
Med-OCT2015-2000m-C282	59,668	CGR	48.1	68	0	—
Med-OCT2015-15m-C165	56,255	CGR	47.7	77	0	SAR116
Med-SEP2014-15m-C135	56,252	CGR	47.5	80	0	SAR116
Med-SEP2014-15m-C146	53,516	CGR	37.6	81	0	SAR116
Med-Ae2-600mDeep-C89	43,595	CGR	35.9	64	0	SAR11
Med-Io17-3500mDeep-C421	42,312	CGR	32.6	59	0	SAR11
Med-OCT2015-15m-C356	41,737	CGR	34.9	56	0	—
MedDCM-SEP2014-C124	40,894	CGR	35.8	52	0	—
MedDCM-SEP2014-C130	40,506	CGR	34.6	53	0	Actinomarina
Med-OCT2015-30m-C129	37,918	CGR	35.1	43	0	—
Med-Io17-3500mDeep-C518	36,171	CGR	30.9	61	0	—
Med-Ae2-600mDeep-C122	35,706	CGR	39.2	122	0	—
Med-Io17-3500mDeep-C537	35,372	CGR	32.1	54	0	Alphaproteobacteria
MedDCM-JUL2012-C215	33,995	CGR	36.6	56	0	—
Med-SEP2014-15m-C363	33,348	CGR	44.6	48	0	Alphaproteobacteria
MedWinter-DEC2013-20m-C766	31,256	CGR	34.9	50	0	—
Med-OCT2015-2000m-C859	30,521	CGR	27.7	38	0	—
MedDCM-SEP2014-C140	39,830	^b^GF	33.1	79	0	SAR11
Med-Ae2-600mDeep-C93	43,016	GF	32.8	59	0	SAR11
MedDCM-JUL2012-C7	154,128	GF	35.9	167	1	Cyanobacteria
Med-OCT2015-15m-C662	30,619	GF	36.2	42	0	SAR11
MAVG-1	166,919	^c^MAVG	39.8	182	2	Cyanobacteria
MAVG-2	157,661	MAVG	34.0	215	2	SAR11
MAVG-3	183,855	MAVG	37.3	215	0	Cyanobacteria
MAVG-4	155,847	MAVG	34.2	208	0	SAR11
MAVG-5	164,624	MAVG	32.7	230	2	SAR11
MAVG-18	46,605	MAVG	34.0	74	0	Actinomarina

^a^CGR: Complete Genome Representative

^b^GF: GenomicFragment

^c^MAVG: Metagenome-Assembled Viral Genomes

In the same way but with contigs from one single sample, we were able to reconstruct a complete genome, MAVG-1, based on the similarity to *Synechococcus* metaG-MbCM1, an already described cultured phage [29] (S4 Fig).

In previous studies where fosmid libraries were used, the length of the insert size (normally 30–40Kb) limited the maximum size of the complete genomes obtained [24,25]. However, using metagenome assembly we have recovered 36 complete genomes with a length ranging from 30 to 196Kb (Table 1) (GC content ranging from 27.7 to 48.8%). In order to analyse the relationships among the complete genomes retrieved here with several phage reference genomes available (400), including those from the Mediterranean uvMED and uvDEEP, we performed an all-versus-all sequence similarity comparison using a previously described methodology [24][25] (S5 Fig). Most of them appear to be related to previously described viruses preying on the major components of the prokaryotic Mediterranean community such as Cyanobacteria, SAR11, SAR116 or Actinobacteria [21,36]. However, we found novel phages for which the assignment of the host was not feasible (Table 1). This large collection of metagenomes and viral contigs provide a different method to obtain complete phage genomes from a natural habitat complementary to viromes in order to advance in the knowledge of the structure and diversity of the viral communities as have been previously described in [17].

In a similar way as we did for the individual reads, we compared the abundance of the VCs between the different filter fractions and sample locations. We took into consideration only those VCs recruiting more than 10 RPKG (Reads per Kilobase of genome per Gigabase of metagenome) of coverage with a similarity >99% in the metagenomic samples to produce the PcoA of S6 Fig. The results showed an even more marked separation than using only individual reads. Both samples belonging to the viral fraction (<0.22 μm) were grouped together but separated from the rest (S6 Fig). The same happened for the particulate fraction samples (5.0–20.0 μm). However, we found four groups for the free-living prokaryotic communities (0.22–5.0 μm) (i) samples belonging to the UP, (ii) LP and DEEP, (iii) DCM samples form the Eastern Mediterranean Sea and (iv) winter MIX samples (S6 Fig). It should be mentioned that the number of contigs obtained is not the same in all the samples (S1A Fig) and in some cases, as in the deep samples, contigs do not reach the minimum value required (10 RPKG) and, as a consequence, they cluster together. However, the same pattern of Fig 1 is repeated and we can see a clear differentiation depending on the filter size fractions.

### Complete genomes and their recruitment from databases

Analysis of the relative abundance of the complete phage genomes in both, cellular (0.22 μm) and viral fractions (<0.2 μm) from the *Tara* Oceans samples, revealed that half of them did not recruit in any station independently of the filter fraction (S7 Fig). Most of them came from deep metagenomes (Med-Ae2-600mDeep, Med-Io17-3500mDeep and Med-OCT2015-2000m) and probably are specific from bathypelagic waters. For this reason, we analysed the abundance also in deep-sea viral metagenomes (POV and Malaspina) but the results did not show any difference, suggesting not only that they are specific of bathypelagic waters but also endemic of the Mediterranean Sea. Another possibility would be that the genomic diversity in bathypelagic waters is higher than in photic regions.

Furthermore, recruitment showed the dominance of cyanophages in metagenomic samples (cellular fraction), consistent with previously observations [26]. However, it is important to point out that phages, mainly cyanophages, often carry auxiliary metabolic genes (AMGs) in order to modify host metabolism during infection. We have noted that the presence of several versions of the same gene coming from bacteria and phages (i.e photosystem II core reaction centre protein D1; PsbA) sometimes breaks the assembly and also overestimate the value of the recruitment but only at values below 90% identity (we have used only 95 and 99% to assess recruitment).

#### Cyanophages

**Fig 4:**
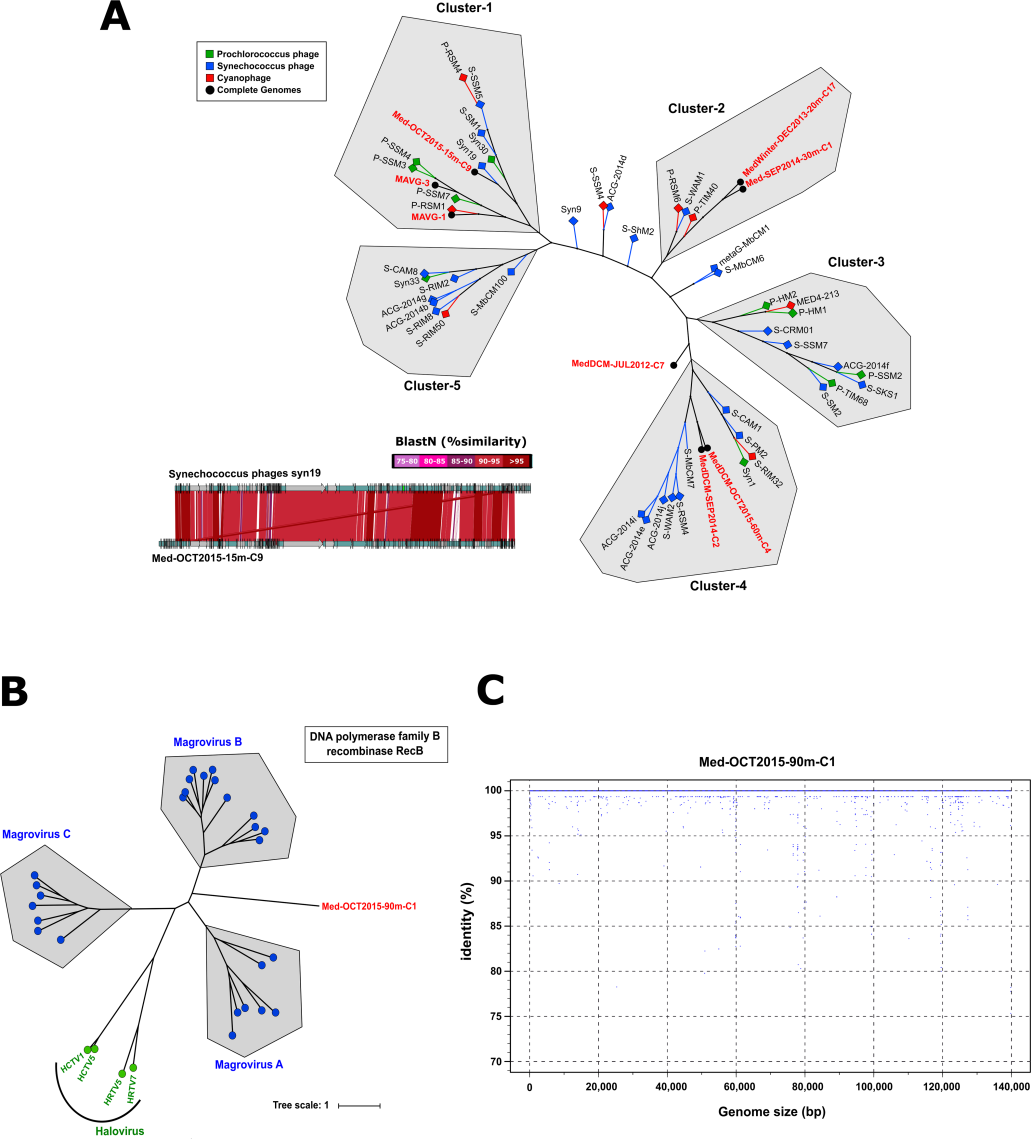
(**A**) Phylogenetic tree of the cyanobacterial phage genomes obtained with 46 reference cyanophage genomes using a concatenation of 33 conserved proteins among them based on sequence similarities against the pVOG database. (**B**) Unrooted maximum likelihood phylogenetic trees of conserved proteins among magrovirus, halovirus and Med-OCT2015-90m-C1 (**C**) Fragment recruitment plot of Med-OCT2015-90m-C1 genome from Med-OCT2015-90m metagenome.

Eight of the complete genomes could be linked to Cyanobacteria (Table 1) based on tRNA gene, all-versus-all sequence tree comparison and the presence of AMGs such as photosystem related genes (*psb*A, *psb*D). In order to examine the phylogenetic distribution among the complete genomes associated to cyanobacteria with 46 reference cyanophage genomes, a maximum likelihood analysis was applied. We used a concatenation of conserved proteins among them based on sequence similarities against the pVOG database [50] (Fig 4A). The core genome was defined by 33 shared-genes, representing *ca*. 16% of the genes in the complete genomes. Fourteen are involved in DNA metabolism (terminase, RecA, DNA polymerase, ATPase, DNA repair/recombination), nine encode structural proteins (tail sheath, neck, prohead core, major head, membrane protein MbpL or base plate wedge subunit) and nine are classified as hypothetical proteins (S3 Table). Five major clusters emerged with a topology including all except one of our genomes, MedDCM-JUL2012-C7, that appears as an outlier. This genome was the smallest and had the lowest GC content (154Kb; GC% 36.0) among complete cyanophages (Table 1). Based on the sequence analysis of the concatenated core genomes, there is no differentiation between *Prochlorococcus* and *Synechococcus* phages among the clusters. However, the reconstructed genome MAVG-3 falls in a separate branch within Cluster-1 with three *Prochlorococcus* phages (P-SSM3, P-SSM4 and P-SSM7) and it is likely to be one itself. Remarkably, we recovered two pairs of identical cyanophage genomes coming from different samples and different years (MedDCM-OCT2015-60m-C4 and MedDCM-SEP2014-C2; MedWinter-DEC2013-20m-C17 and Med-SEP2014-30m-C1). The first two, MedDCM-OCT2015-60m-C4 and MedDCM-SEP2014-C2, the largest assembled genomes with 196Kb, also represented a new cyanophage group within Cluster-4. However, some of them clustered together with known cyanophage isolates, for example, MedDCM-OCT2015-15m-C9 clustered with *Synechococcus* phages syn19 isolated from the North Atlantic in 1990 [43]. Both genomes were similar (*ca*. 90%ANI) and completely syntenic with only small variable regions (Fig 4A, inset). Although both phages contained six tRNA genes, only one was 100% identical, while the others were very different. These could help them to have a wide range of hosts with different tRNA sequences. A similar case also happened with MAVG-1 and cyanophage P-RSM1 isolated from the Red Sea but in this example the similarity was less than 90%.

#### Pelagiphages

Based on the all-versus-all comparison, terminase phylogeny and the comparison with previous studies [24,25], nine of the complete genomes could be classified as new SAR11 infecting phages (pelagiphages) (Table 1). Some of them similar to the only four pelagiphages isolated from seawater samples using *Ca*. Pelagibacter ubique HTCC1062 as a host [51]. Cluster-153 contained two CGRs of 43Kb, Med-Ae2-600mDeep-C93 and Med-Io17-3500mDeep-C421, with an ANI of ca. 96% (coverage 62%) and seem to be related to the cultured *Pelagibacter* podovirus HTVC019P [51] and the uvMED genome AP013545 [24] (S8A Fig). Despite the fact that these new genomes were obtained from deep metagenomes (650 and 3,500m, respectively), genome organization comparison with DCM or surface reference genomes showed that synteny was well conserved. However, we found a gap or flexible region (7Kb) among them containing three genes encoding a tryptophanyl-tRNA synthetase, putative internal virion protein and a pesticin domain protein (S8A Fig). Single nucleotide polymorphism (SNP) analyses between these two CGRs showed the major variation in two genes encoding a phage tail fiber protein, known to be involved in host range specificity [23] and a DNA methyltransferase. Recruitment showed that these new group of pelagiphages is not restricted to the deep waters of the eastern Mediterranean since they also appear in the station Malaspina viral metagenome MSP-114 (95% identity) from the Pacific Ocean at a depth of 4,000m. In fact, a new clade of SAR11 representatives, subclade Ic, which could be potential hosts of these phages, has been found exclusively in deep waters [52].

Contrastingly, MedDCM-SEP2014-C140 obtained from the DCM at the Western Mediterranean recruited in the photic zone (UP and DCM) along the whole Mediterranean Sea in both (cellular and viral) fractions (S9 Fig) and also in one station in the South Atlantic Ocean. This phage showed synteny and nucleotide sequence similarity (<70%) to uvDEEP genome KT997865, obtained from 3,000m deep in the Ionian Sea tentatively predicted to infect SAR11 representatives [25]. A metaviromic island [53] was found in all of the datasets comprising a gene encoding a phage tail tape measure protein. This protein is critical for infection since determines the tail length and allows the DNA injection into the cell [54].

Med-OCT2015-15m-C662 could be also classified as a new pelagiphage since it displayed high sequence similarity as well as an unique integrase with members of G15 previously described [24] related to HTVC010P (S8B Fig). Recruitment showed that Med-OCT2015-15m-C662 was found restricted only to surface waters in two viromes from the Ionian Sea (S8B Fig). Comparison with the closely related genomes and recruitment showed a variable region containing four genes, but no function related to host recognition could be inferred. The other five complete genomes (Med-OCT2015-15m-C16, Med-OCT2015-15m-C18, Med-Io7-70mDCM-C14, MAVG-2, -4 and -5) with a larger size than the previous between 138 and 164Kb (GC content range 32.7 to 39.37%) were classified based on whole genome comparison and tRNA genes with *Pelagibacter* phage HTVC008M (S5 Fig and Table 1) [51].

#### SAR116 phages

We also detected three CGRs of putative SAR116 clade phage genomes encompassed in two different clusters (Table 1). One of the clusters containing CGRs Med-OCT2015-15m-C165 and sept14-15m-C135 showed high GC content (*ca*. 47.6%) similar to members of the SAR116 clade while the other had only 37.6% GC content. Despite the fact that the first SAR116 phage, HMO-2011, is considered one of the most abundant cultured marine viruses [47], only one of the CGRs (Med-SEP2014-15m-C146) was detected in three *Tara* station viromes from surface samples and only in different locations of the Ionian Sea (S7 Fig).

#### Marine Euryarchaeota group II (magrovirus)

CGR Med-OCT2015-90m-C1 (140Kb) contained two tRNA sequences, with one having an exact match to an archaeal tRNA-Arg-TCT gene found in the Marine Euryarchaeota group II Thalassoarchaea [35]. This finding classified this CGR as a new magrovirus (MArine GROup II viruses) [26]. Three distinct magrovirus groups comprising 26 complete genomes assembled from metagenomic datasets have been recently described [26]. On top of that, two conserved proteins (DNA polymerase family B and recombinase RecB) were found conserved in the CGR, all the magroviruses described and several haloviruses selected as references (based on the pVOG database; [50]). A phylogenetic tree clearly placed this novel head-tailed archaeal virus of about 140Kb as a new group within these magrovirus (Fig 4B). We have analysed the global distribution of this phage in all viromes and metagenomes used in this study and also from the *Tara* Oceans and Malaspina expeditions with a threshold of 70% identity. Interestingly, we only found reads at more than 99% identity in Med-OCT2015-75m and Med-OCT2015-90m metagenomes showing that we recovered a specific lineage endemic from the LP waters of the Western Mediterranean (Fig 4C).

#### Virophage

In addition, from the Med-OCT2015-2000m metagenome we assembled a virophage-like genome (Med-OCT2015-2000m-C859). As far as we know, only 18 genomes have been described so far in a broad range of habitats worldwide [55] and this is the first report of a complete virophage from deep seawater (2,000m). Virophages are considered a special group of viruses that parasitize giant viruses of the family Mimiviridae [55]. Although they are obligate parasites, little is known about their ecological relevance, replication or dynamics. The genome is composed of 30,521bp and contains 35 ORFs including the virophage core genes encoding the major and minor capsid proteins, packaging ATPase, cysteine protease and the DNA replication protein (S10A Fig). These core genes were used to reconstruct a phylogenetic tree. As shown in S10B Fig, Med-OCT2015-2000m clustered together with YSLV5, a virophage identified in Yellowstone Lake through metagenomic analyses [56], despite their differences in GC content (27.7 versus 51.1%).

### Eurybathic or stenobathic

With the large collection of marine metagenomes and viromes available, it is possible to evaluate not only the most abundant and widespread VCs but also their distribution in the water column. Furthermore, datasets obtained from different years from the same location can be used to detect patterns of temporal variation and evolution. We have used recruitment of metagenomic reads to elucidate possible patterns of distribution of these phages in nature.

The majority of the global marine viral metagenomic studies [15] are focused on surface samples considering the photic zone as a homogeneous compartment and taking into account only the differences between the photic and aphotic zone. To investigate the vertical distribution of the VCs throughout the water column, we used recruitment of metagenomic reads from a fine-scale metagenomics profile (every 15m) in a stratified and mature (early autumn) Western Mediterranean water column [36]. Based on the vertical distribution, phages can be categorized into two types: eurybathic (broad depth distribution) and stenobathic (restricted to narrow depth range). We took into consideration only those VCs recruiting more than 10 RPKG of coverage with a similarity >95% in the metagenomic profile. We found 227 out of 927 VCs abundant in at least one of the metagenomes, although none of them were detected in deeper waters (1,000 and 2,000m). It is remarkable that a large number of the VCs (*ca*. 89%) appear to be found exclusively in one single specific depth or two contiguous depth metagenomes (S11 Fig). Moreover, this distribution was more marked in the UP and DCM where typically genomes appeared at one or the other while *ca*. 50% of the VCs found beyond the DCM were present in both depths (75 and 90m) (S11 Fig). This stenobathic character is consistent with the narrow depth distributions found in the analysis of the prokaryotic fraction in these metagenomes [36] and suggest that most of the phages have a specialized host range (not generalist). Only two singletons (MedDCM-SEP2013-LF-C8 and Med-OCT2015-30m-C1728) that appear related to the pelagiphage HTVC008M recruited in all the photic metagenomes and could be considered eurybathic phages. Regarding the remaining 11% of the VCs that recruit in more than one of the metagenomes they did not show any decrease with depth, indicating that there is no vertical viral transport in sinking particles as was previously hypothesized [57].

### Endemic or widespread

To assess the abundance and distribution of the novel VCs we performed fragment recruitment analysis by comparing each VC to that of numerous metagenomes from *Tara* Oceans datasets (cellular and viral fraction). We considered only those VCs recruiting more than 10 RPKG and present in at least two stations. Besides, we have used two very restrictive nucleotide identity thresholds, 95 and 99%.

**Fig 5:**
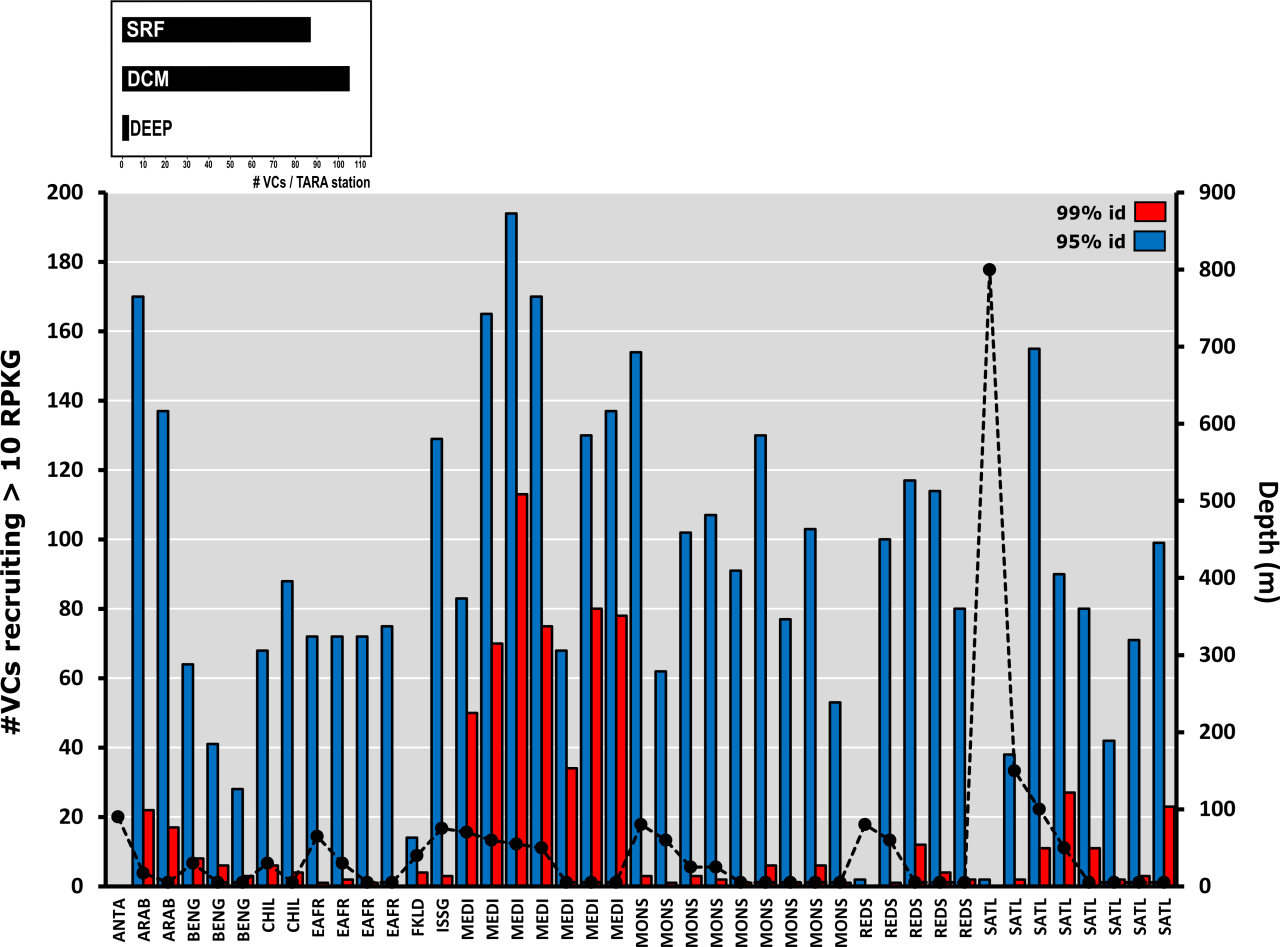
Recruitment of the Viral clusters (VCs) and singletons that recruits more than 10 RPKG (Reads per Kilobase of genome and Gigabase of metagenome) in at least two stations of the *Tara* Oceans viromes. Left axis and dotted line indicates depth of sample. Upper panel shows the normalized value of total VCs by the number of stations belonging to SRF (surface) DCM (deep chlorophyll maximum) and DEEP (deep). Region abbreviations are as follows: ANTA, Antarctic Province; ARAB, Northwest Arabian Sea Upwelling Province; BENG, Benguela Current Coastal Province; CHIL, Chile-Peru Current Coastal Province; EAFR, Eastern Africa Coastal Province; FKLD, Southwest Atlantic Shelves Province; ISSG, Indian South Subtropical Gyre Province; MEDI, Mediterranean Sea Black Sea Province; MONS, Indian Monsoon Gyres Province; REDS, Red Sea; SATL, South Atlantic Gyral Province.

At 99% of identity, VCs were found mainly at their habitat of origin (Mediterranean Sea) in both fractions, reasserting the endemicity at this level of similarity of these genomes (Fig 5 and S12 Fig). We found some exceptions, a peak in the metagenomic sample from the station TARA_004 a North Atlantic Ocean station, but from the region next to the Gibraltar Strait (the connection between Mediterranean Sea and Atlantic Ocean) and also two samples TARA_132 and 133 located in the North Pacific Ocean. The prevalence of these VCs might reflect similar conditions since these stations were located at similar latitudes than the Mediterranean Sea. However, we did not find the same abundance in similar regions in the Atlantic Ocean, probably due to the stratification of the water column that is permanent in these two Pacific samples (like in the Mediterranean during the time of sampling) while the Atlantic samples were collected during the mixed period.

On the other hand, when identity was shifted to 95%, results indicated that these viruses are globally widespread with the exceptions of polar latitudes and mesopelagic samples (from 280 to 800m) in both fractions (Fig 5 and S12 Fig). We found that 514 of the 927 VCs (55.4%) recruited in more than two metagenomic or viromic datasets. In total, 208 were abundant in both fractions, 190 were reported only in the cellular fraction and 116 in the viral fraction. While pelagiphages were the most widely distributed group in the viral fraction, cyanophages were dominant in the cellular fraction (as found before [26]). The VC MedWinter-DEC2013-20m-C2965 classified as a pelagiphage was the one found in the largest numbers of *Tara* stations (widespread) while the highest absolute recruitment value found for a contig was that of the CGR MedWinter-DEC2013-20m-C17, described as a new cyanophage, which recruited more than 480 RPKG in TARA_007 station near its isolation place. On the other hand, contigs recovered from deep metagenomes did not recruit in any *Tara* metagenome or virome (most of the datasets were collected in the photic zone). Using the same parameters, we have analysed the abundance and distribution of these novel VCs also in the deep-sea viral metagenomes. While at 99% of identity only a couple of VCs recruited in all the Malaspina and POV stations when we moved to the population cut-off (95%), the numbers did not change excessively as in the previous case with the *Tara* dataset. Twelve VCs were found in Malaspina and only four in POV, most of them recovered from the Med-Io17-3500mDeep metagenome emphasizing their truly bathypelagic nature. The low recruitment in the deep viromes in comparison with the ones of the photic zone can be due to the special conditions of the deep Mediterranean Sea which is much warmer (>13°C) and contain lower concentrations of inorganic nutrients N and P than waters of similar depth in open oceans. This special situation might allow the persistence of specific microbial communities adapted to aphotic regions [31] and hence of the viruses associated with them. These data suggest that, at least in the Mediterranean Sea, there is a clear evidence of a local viral distribution and diversity (more marked in deep waters).

## Materials and methods

### Sampling, sequencing, assembly and annotation

Eleven seawater samples were collected at different depths, filter pore size and season (stratified or mixed) during consecutive years in the Western Mediterranean. Metadata of the samples are summarized in S1A and S1B Fig. Samples from 2012 to 2015 (MedDCM-JUL2012, MedDCM-SEP2013, MedDCM-SEP2013-LF, MedWinter-DEC2013-20m, Med-SEP2014-15m, Med-SEP2014-30m, MedDCM-SEP2014, MedWinter-JAN2015-20m, MedWinter-JAN2015-20m-LF, MedWinter-JAN2015-20m, MedWinter-JAN2015-20m-LF, MedDCM-SEP2015_HS) [34,35,38] [36] were recovered at 20 nautical miles off the coast of Alicante (38.06851°N, 0.231994°W; bottom depth of 200 m). Additionally, in 2015 another nine samples (Med-OCT2015-15m, Med-OCT2015-30m, Med-OCT2015-45m, Med-OCT2015-60m, Med-OCT2015-75m, Med-OCT2015-90m, Med-OCT2015-1000m and Med-OCT2015-2000m) [36] were collected at approximately 60 nautical miles off the coast of Alicante (37.35361°N, 0.286194°W). Five more samples coming from different locations in the Eastern Mediterranean were used [25]. Med-Io16-70mDCM, Med-Io7-77mDCM and Med-Io17-3500mDeep recovered from the Ionian Sea, at depths of 70, 77 and 3500 meters, respectively. Finally, two samples collected from the Aegean Sea, at 75m (Med-Ae1-75mDCM) and at 600m deep (Med-Ae2-600mDeep) were also included in the analysis [25,34].

All seawater samples were sequentially filtered on board through 20 μm nylon mesh and 5 and 0.22 μm pore size polycarbonate filters (Millipore). All filters were immediately frozen on dry ice and stored at -80°C until processing. DNA extraction was performed from 0.22 and 5 μm filters as previously described [58]. Metagenomes collected on September and October 2015 were sequenced using Illumina Hiseq-4000 (150bp, paired-end read) (Macrogen, Republic of Korea). The remaining metagenomes were sequenced using Illumina Hiseq-2000 (100bp, paired-end read) (BGI, Hong Kong) obtaining sequence data in a range between 15 and 20 Gb. Individual metagenomes were assembled using IDBA-UD [59]. The resulting genes on the assembled contigs were predicted using Prodigal [60]. tRNA and rRNA genes were predicted using tRNAscan-SE [61], ssu-align [62] and meta-rna [63]. Predicted protein sequences were compared against NCBI NR, COG [64] and TIGRFAM [65] databases using USEARCH6 [66] for taxonomic and functional annotation. GC content was calculated using the GeeCee program from the EMBOSS package [67]. Proteins were clustered using CD-HIT [68] at 60% sequence identity and > 80% alignment on the shorter sequence.

### Virome sampling and sequencing

One of the viromes (MedDCM-Vir) was obtained from the DCM of the Mediterranean Sea (65 m deep) on August 29th, 2011. DNA was amplified by MDA and sequenced by Illumina to provide nearly 18 Gb of sequence data as was described in [24]. The other viromic sample (MetaVir-2013) was collected from the Mediterranean DCM at 55m (38.06851°N, 0.231994°W; bottom depth of 200 m) on 6 September 2013. Sample was processed in the same way as the other virome MedDCM-Vir [24]. However, the amount of DNA obtained was sufficient to sequence and it was not necessary to use any amplification treatment. Phages were concentrated using tangential flow filtration (TFF) with a 30 kD polyethersulfone membrane from Vivaflow (VF20P2). The resulting phage concentrate was ultracentrifuged (Optima XL 1000K Ultracentrifuge, Beckman) for 1 h at 4°C using a Type 70 Ti rotor (Beckman) at 30,000 rpm (92,600 g). The pellet was treated with 2.5 units DNase I at 37°C for 1 hr, and 70°C for 10 min to remove bacterial DNA. DNA was sequenced using Illumina Hiseq-2000 (100bp, paired-end read) (BGI, Hong Kong).

### Viral contigs and host prediction

In order to confirm the viral origin of the contigs we performed a manual inspection based on the resemblance to known phages similar to methods that have been previously described [24,25]. Complete genomes were identified searching for overlapping sequences in the 3′ and 5′ region (at least 30bp). These contigs were clustered using an all-versus-all BLASTN comparison with a cut-off of 90% sequence identity and 20% coverage. We have used different host prediction approaches to identify the putative host of the viral contigs. These methods have been previously described [24] and include tRNA matches, CRISPR spacers, presence of AMGs, all-versus-all comparison and terminase phylogeny. Furthermore, all the contigs were annotated and assigned if a majority of genes gave best BLAST hits against the NR database (>75% nucleotide identity and >50% coverage) to the same phage.

### Taxonomic read analysis

Subsets of 20 million reads ≥ 50bp (where applicable) were taxonomically classified against the NR database using DIAMOND [69] with a minimum of 50% identity and 50% alignment. The resulting alignment was later analyzed with MEGAN6 Community Edition [70], and both Unweighted Pair Group Method with Arithmetic Mean (UPGMA) taxonomic tree and canonical correspondence analysis (CCA) were inferred with the cluster analysis option and a Bray-Curtis ecological distance matrix.

### Metagenomic read recruitments

The abundance and distribution of the VCs obtained in this study were performed using recruitment against the complete dataset of *Tara* Oceans metagenomes [71] and viromes [15], metagenomes from this study (S1 Fig and Table 1) and also deep-sea viral metagenomes from the Malaspina expedition [45] and POV dataset [16]. Metagenomic recruitment of the reads were compared using BLASTN [72] and hits obtained were used to compute the RPKG (reads recruited per Kb of genome per Gb of metagenome) values that provide a normalized number comparable across various metagenomes.

### Complete phage genome comparison

Complete phage genomes were compared to several well-classified Caudovirales (Podoviridae, Myoviridae and Siphoviridae) reference phages downloaded from the NCBI, in addition to known marine phage genomes and previously published marine phages (APXXX and KTXXX) [24,25]. Dice coefficient between genomes was computed from summed calculated TBLASTX scores as previously reported [24]. This metric was transformed to a dissimilarity metric and values were log10 converted to reduce the distance between extreme values. A neighbour joining tree was constructed from the complete distance matrix using the phangorn package [73] in R and formatted in Dendroscope [74]. SNPs between phage genomes were identified using nucmer program in the MUMmer3+ package [75].

## Supporting information

S1 Fig (**A**) Summary of sampling parameters and assembly statistics of the raw reads obtained from metagenomes. (**B**) Site and depth profiles of the samples.
(PDF)

S2 Fig Comparison between uvMED and uvDEEP with metagenomic contigs obtained in this study. (PDF)

S3 Fig Terminase phylogeny. A maximum likelihood phylogenetic tree of the four major types of phage terminase large-subunit domains is shown.
(PDF)

S4 Fig Reconstruction of MAVG-1 based on the similarity to *Synechococcus* metaG-MbCM1. (PDF)

S5 Fig All-versus-all sequence similarity comparison of CGRs with several marine phage reference genomes available. (PDF)

S6 Fig PCoA based on recruitment of the Viral clusters between the different filter fractions and sample locations. Samples highlighted in blue and red correspond to samples obtained from the 5–20μm and <0.22μm filters, respectively.
(PDF)

S7 Fig Recruitment at 99% identity of the complete viral genomes along *Tara* stations. Recruitments in viromic samples are showed on the left panel. On the right, recruitments in the metagenomic samples are represented.
(PDF)

S8 Fig (**A**) Two CGRs in comparison to the cultivated pelagiphage HTVC019P and the uvMED genome AP013545. (**B**). Genome comparison between Med-OCT2015-15m-C662 and members of the previously described G15 group related to pelagiphage HTVC010P.
(PDF)

S9 Fig Genome comparison between MedDCM-SEP2014-C140 and uvDEEP genome KT997865 previously described as a phage infecting SAR11 representatives. (PDF)

S10 Fig (**A**) Complete genome of Med-OCT2015-2000m-C859, homologous genes in other virophages are labeled. (**B**) Maximum-likelihood-based phylogenetic analysis of the concatenation of seven shared amino acid sequences with other already described virophages.
(PDF)

S11 Fig Relative abundance of the viral cluster and singletons measured by recruitment (RPKG) from the different depth metagenomes. We took into consideration only those Viral clusters recruiting more than 10 RPKG.
(PDF)

S12 Fig Recruitment of the Viral clusters and singletons that recruits more than 10RPKG in at least two stations of the Tara Oceans metagenomes. Left axis indicates depth of sample.
(PDF)

S1 Table Phage Orthologous Groups assigned to one representative of each protein viral cluster. (XLSX)

S2 Table Complete list of all phage contigs described in this study. (XLSX)

S3 Table pVOG categories associated to the 33 conserved proteins among the 54 cyanophage genomes compared. (XLSX)
