# Supplementary material for: Genome diversity of marine phages recovered from Mediterranean metagenomes: Size matters
Source: PLoS Genet. 2017 Sep 25;13(9):e1007018. doi: 10.1371/journal.pgen.1007018 (PMC5628999; doi:10.1371/journal.pgen.1007018)

A

Western Mediterranean	Sampling Data	Size fraction	Location	Depth (m)	Sample classification	Prokaryotic Contigs (>10kb)	Viral Contigs (#)	Largest (Kb)	GC content	Reference
**Med-OCT2015-15m**	10/15/2015	5-0.22 µm	Spain: off the coast of Alicante	15	UP	4,648	117	175	33.9	Haro-Moreno et al. 2017
**Med-OCT2015-30m**	10/15/2015	5-0.22 µm	Spain: off the coast of Alicante	30	UP	2,198	166	164	35	Haro-Moreno et al. 2017
**MedDCM-OCT2015-60m**	10/15/2015	5-0.22 µm	Spain: off the coast of Alicante	60	DCM	2,360	51	196	34.1	Haro-Moreno et al. 2017
**Med-OCT2015-75m**	10/15/2015	5-0.22 µm	Spain: off the coast of Alicante	75	LP	2,789	62	148	34.3	Haro-Moreno et al. 2017
**Med-OCT2015-90m**	10/15/2015	5-0.22 µm	Spain: off the coast of Alicante	90	LP	2,556	47	80	33.3	Haro-Moreno et al. 2017
**Med-OCT2015-1000m**	10/16/2015	5-0.22 µm	Spain: off the coast of Alicante	1,000	DEEP	1,807	9	19	39.4	Haro-Moreno et al. 2017
**Med-OCT2015-2000m**	10/16/2015	5-0.22 µm	Spain: off the coast of Alicante	2,000	DEEP	4,699	8	59	39.4	Haro-Moreno et al. 2017
**MedDCM-JUL2012**	06/20/2012	5-0.22 µm	Spain: off the coast of Alicante	75	LP	1,625	16	50	36.5	Martin-Cuadrado et al, 2015
**MedDCM-SEP2013**	09/06/2013	5-0.22 µm	Spain: off the coast of Alicante	55	DCM	720	40	68	37.2	Martin-Cuadrado et al, 2015
**MedDCM-SEP2013-LF**	09/06/2013	20-5 µm	Spain: off the coast of Alicante	55	DCM	13	5	19	33.1	López-Pérez et al, 2016
**MedWinter-DEC2013-20m**	13/12/2013	5-0.22 µm	Spain: off the coast of Alicante	20	MIX	452	49	188	35.8	This study
**Med-SEP2014-15m**	09/12/2014	5-0.22 µm	Spain: off the coast of Alicante	15	UP	3,025	87	142	37.1	This study
**Med-SEP2014-30m**	09/12/2014	5-0.22 µm	Spain: off the coast of Alicante	30	UP	2,085	101	188	36	This study
**MedDCM-SEP2014**	09/12/2014	5-0.22 µm	Spain: off the coast of Alicante	60	DCM	1,937	110	196	36.8	Haro-Moreno et al. 2017**
**MedWinter-JAN2015-20m-LF**	01/27/2015	20-5 µm	Spain: off the coast of Alicante	20	MIX	424	12	34	40.2	López-Pérez et al, 2016
**MedWinter-JAN2015-Coastal**	01/27/2015	5-0.22 µm	Spain: off the coast of Alicante	15	MIX	873	8	22	39.5	This study
**MedWinter-JAN2015-20m**	01/27/2015	5-0.22 µm	Spain: off the coast of Alicante	20	MIX	869	0	0	0	López-Pérez et al, 2016
**MedWinter-JAN2015-80m**	01/27/2015	5-0.22 µm	Spain: off the coast of Alicante	80	MIX	885	13	45	39.1	This study
**MedDCM-SEP2015_HS**	09/12/2015	5-0.22 µm	Spain: off the coast of Alicante	75	DCM	4,538	138	163	34.8	Haro-Moreno et al. 2017
**Eastern Mediterranean**	**Sampling Data**	**Size fraction**	**Location**	**Depth (m)**	**Sample classification**	**Prokaryotic Contigs (>10kb)**	**Viral Contigs (#)**	**Largest (Kb)**	**GC content**	**Reference**
**Med-Io7-77mDCM**	10/01/2010	5-0.22 µm	Italy, Ionian Sea	77	DCM	2,340	109	145	36.5	Mizuno et al. 2016
**Med-Io16-70mDCM**	10/02/2010	5-0.22 µm	Italy, Ionian Sea	70	DCM	340	77	91	35.2	Mizuno et al. 2016
**Med-Io17-3500mDeep**	10/02/2010	5-0.22 µm	Italy, Ionian Sea	3,500	DEEP	2,999	34	55	39.1	Mizuno et al. 2016
**Med-Ae1-75mDCM**	10/03/2010	5-0.22 µm	Greece, Aegean Sea	75	DCM	371	30	61	35.3	Mizuno et al. 2016
**Med-Ae2-600mDeep**	10/03/2010	5-0.22 µm	Greece, Aegean Sea	600	DEEP	1,145	34	94	38.7	Mizuno et al. 2016

B

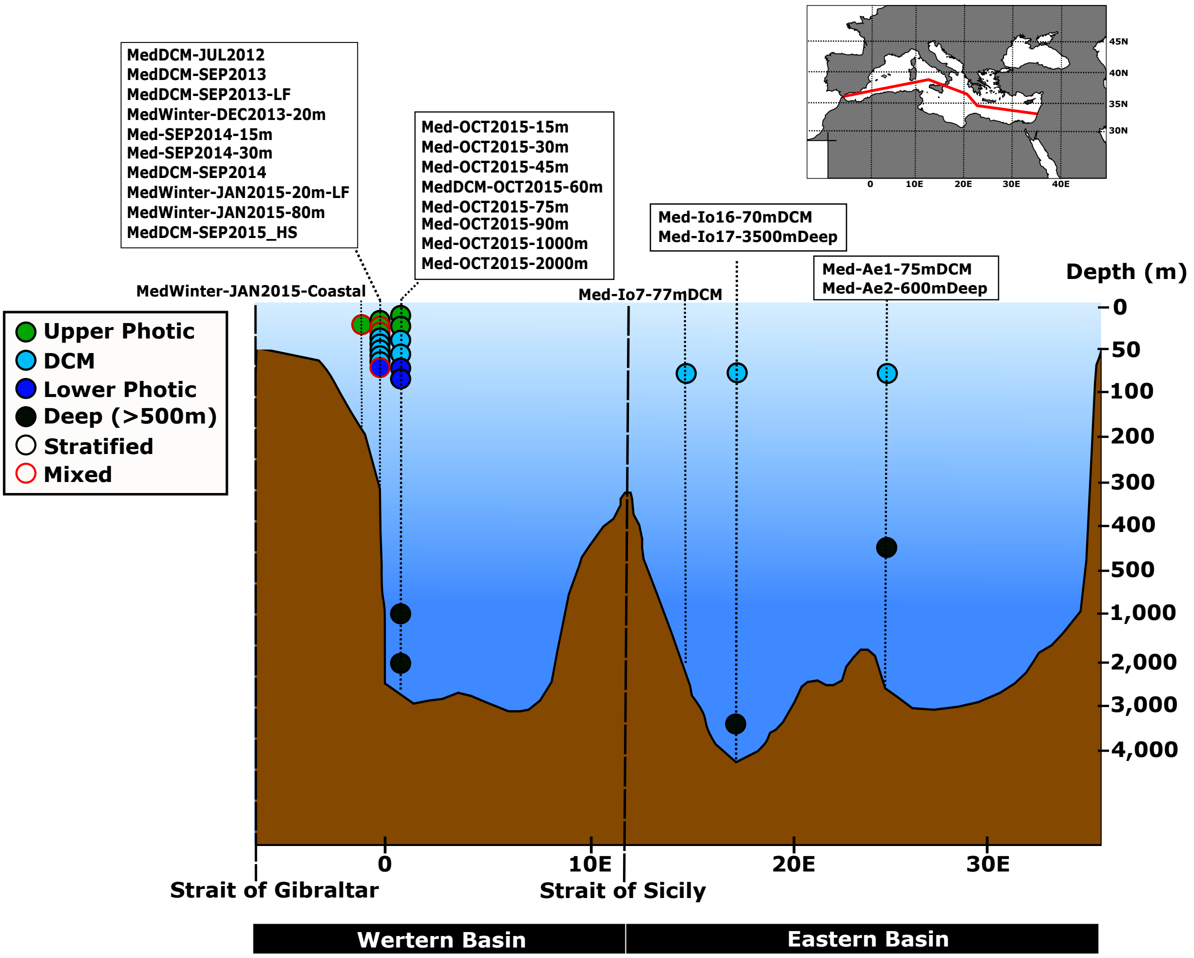

Supplement: S1 Fig — (A) Summary of sampling parameters and assembly statistics of the raw reads obtained from metagenomes. (B) Site and depth profiles of the samples. (PDF) [file pgen.1007018.s001.pdf]
