# Supplementary figures and images for: Genome diversity of marine phages recovered from Mediterranean metagenomes: Size matters
Source: PLoS Genet. 2017 Sep 25;13(9):e1007018. doi: 10.1371/journal.pgen.1007018 (PMC5628999; doi:10.1371/journal.pgen.1007018)

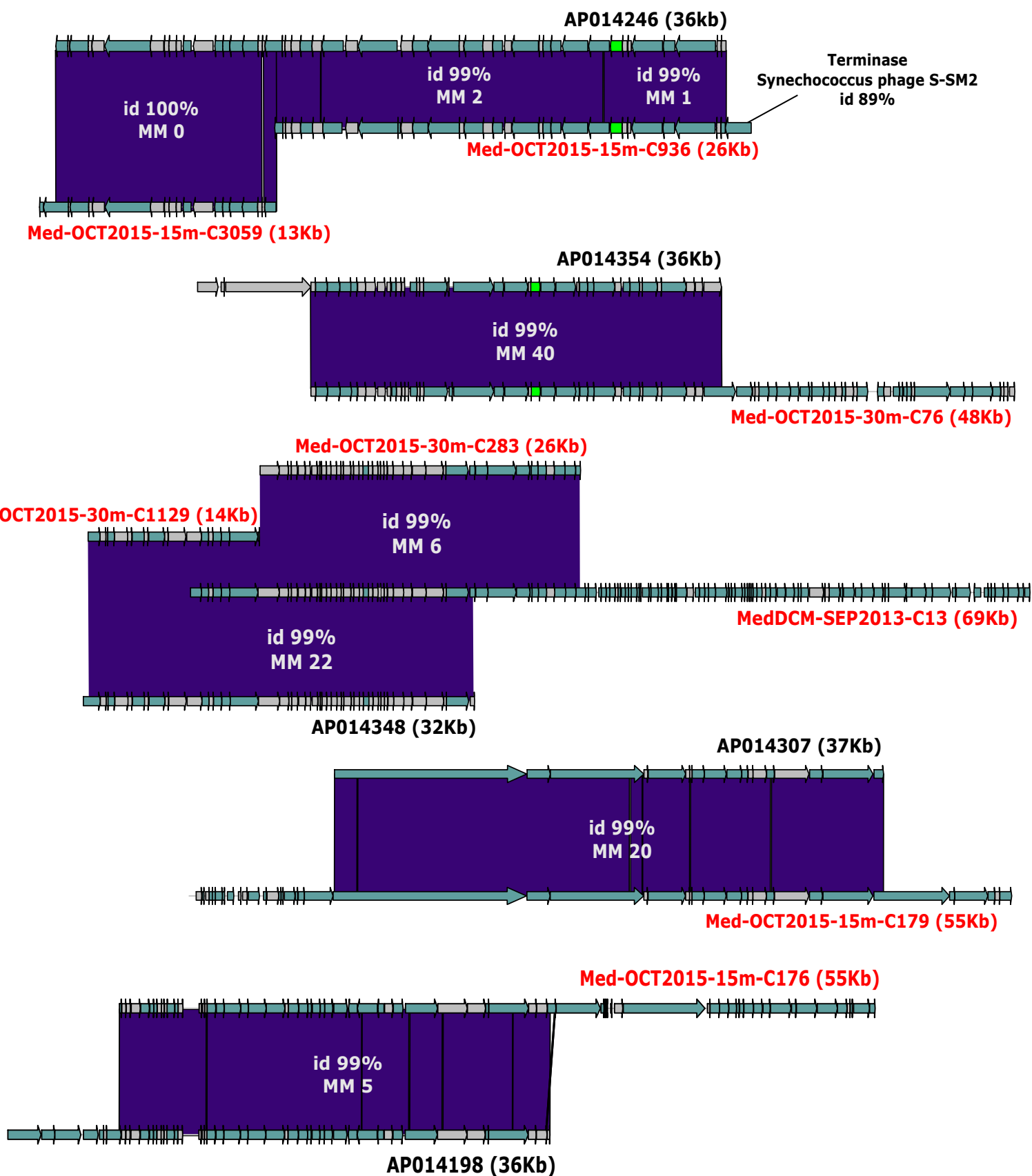

Supplement: S2 Fig — (PDF) [file pgen.1007018.s002.pdf]

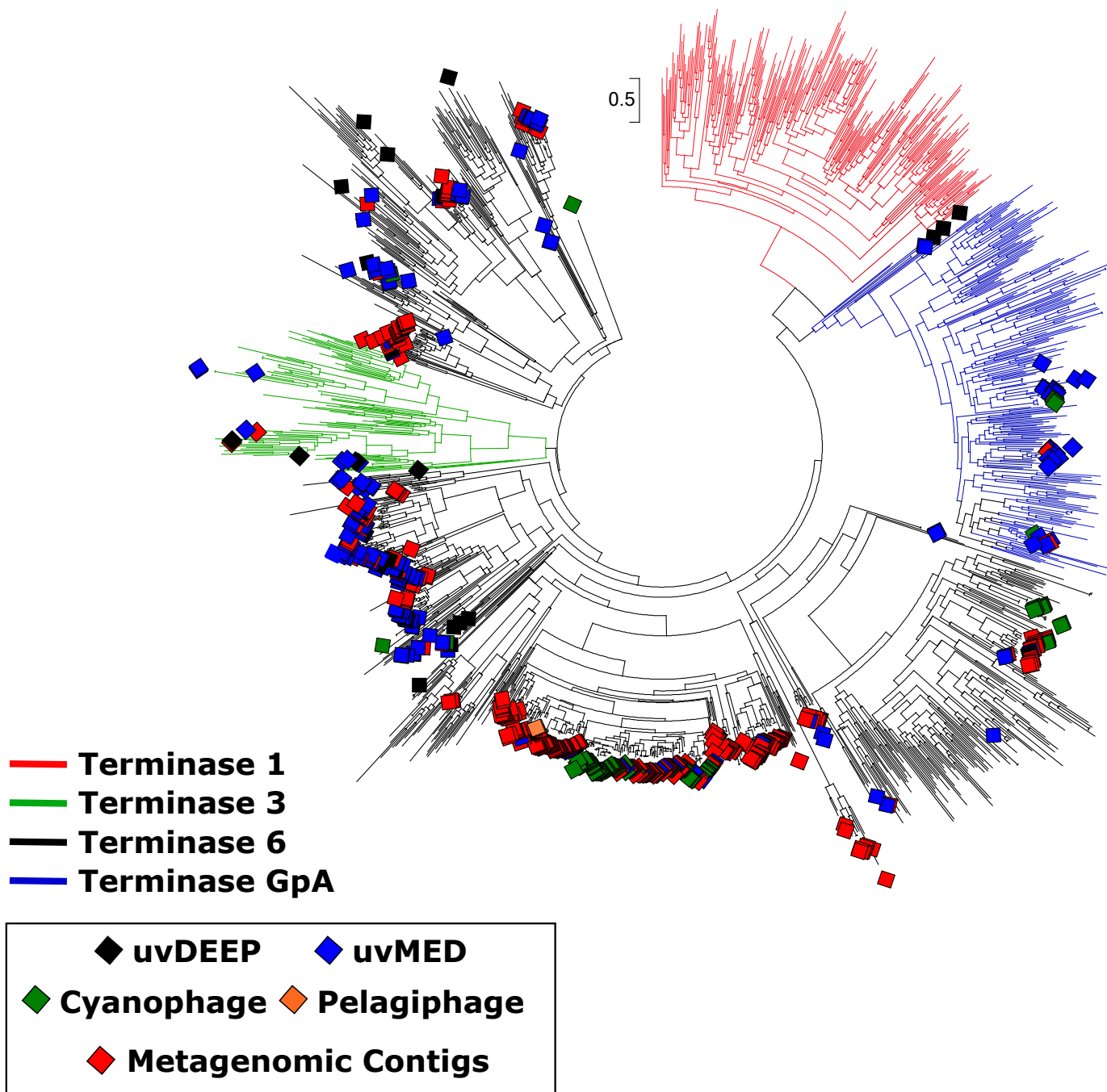

Supplement: S3 Fig — A maximum likelihood phylogenetic tree of the four major types of phage terminase large-subunit domains is shown. (PDF) [file pgen.1007018.s003.pdf]

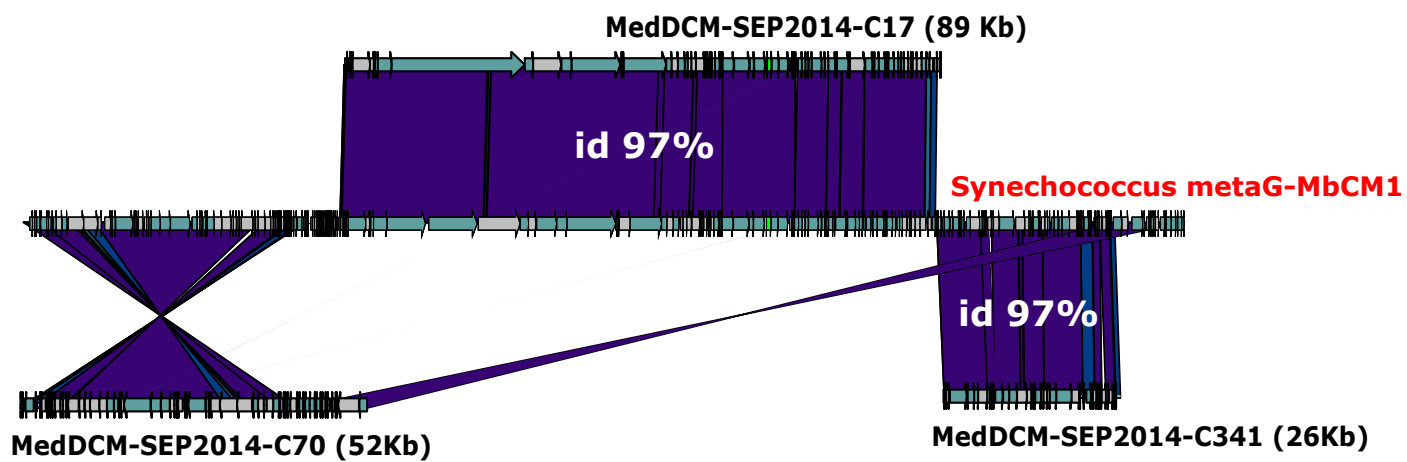

Supplement: S4 Fig — (PDF) [file pgen.1007018.s004.pdf]

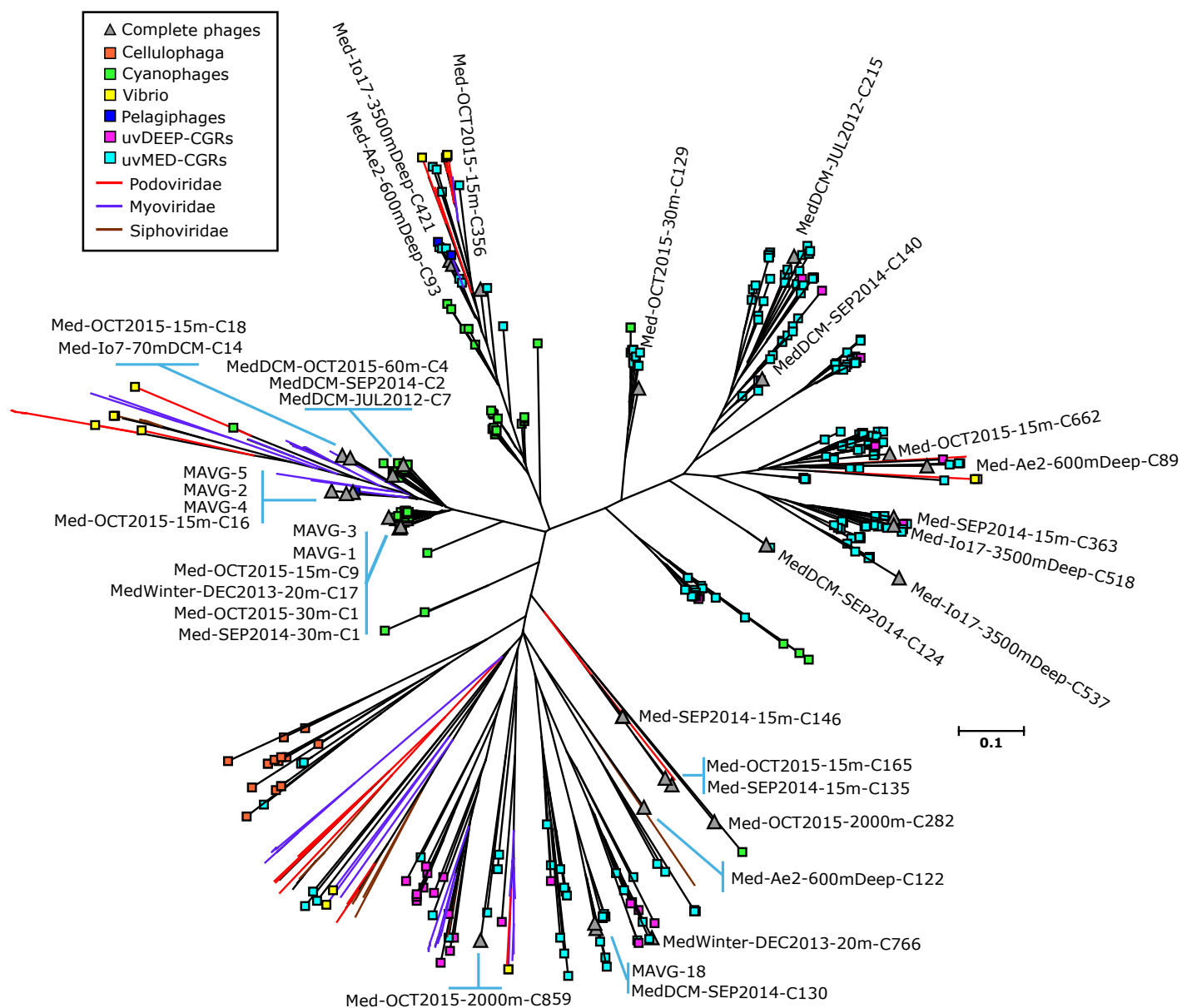

Supplement: S5 Fig — (PDF) [file pgen.1007018.s005.pdf]

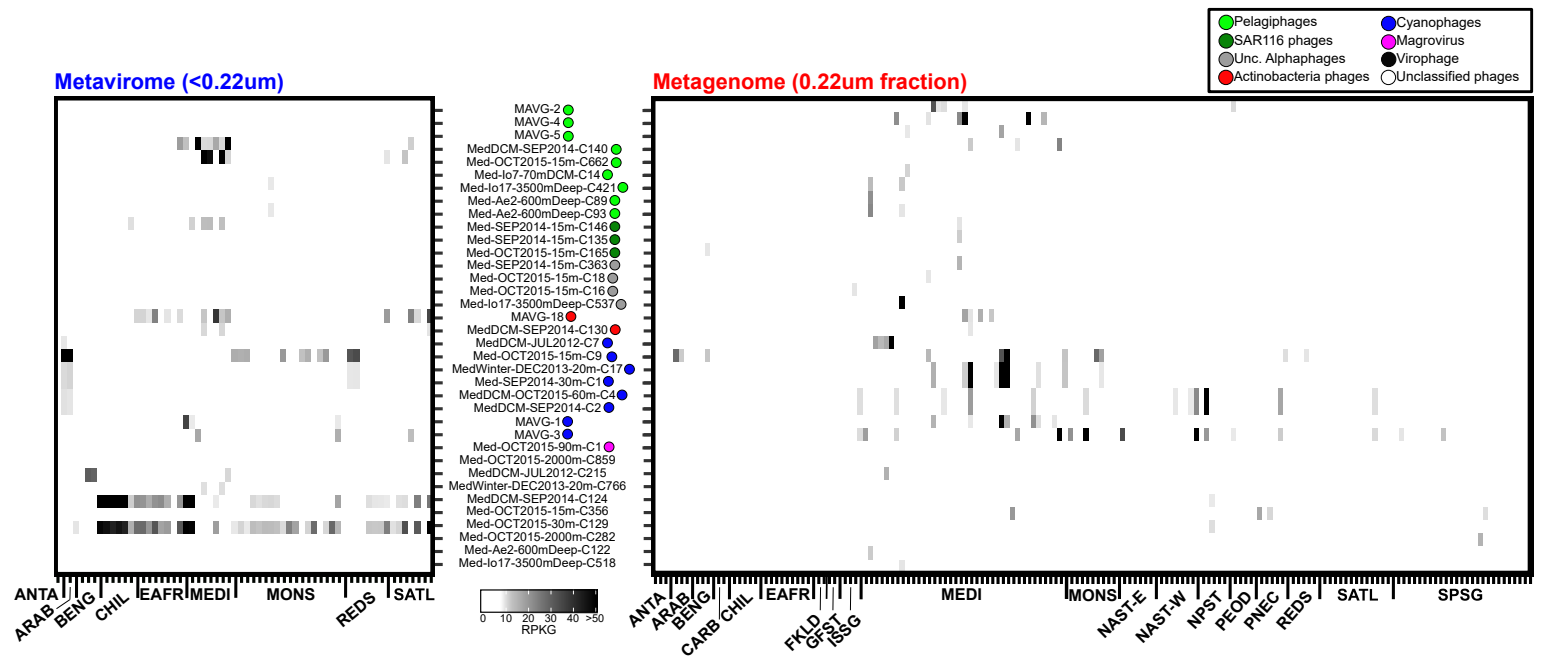

Supplement: S7 Fig — Recruitments in viromic samples are showed on the left panel. On the right, recruitments in the metagenomic samples are represented. (PDF) [file pgen.1007018.s007.pdf]

**A**

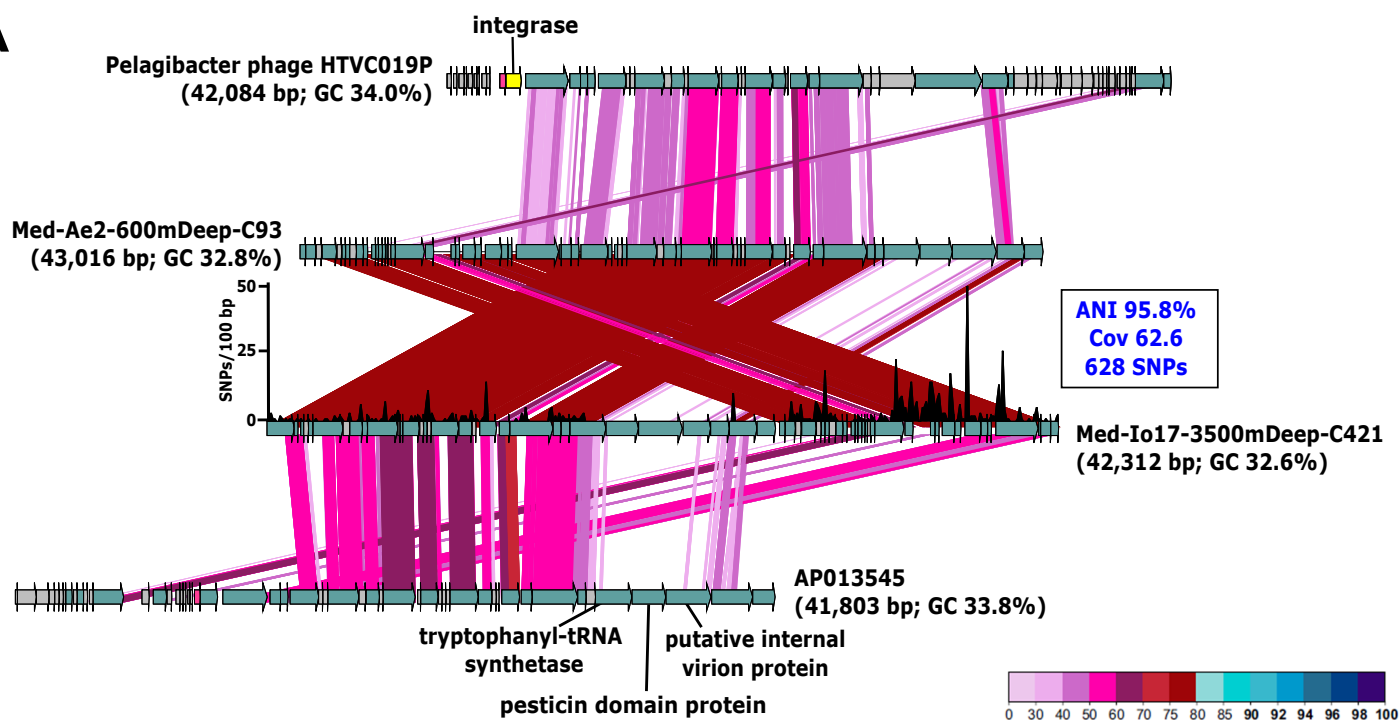

**B**

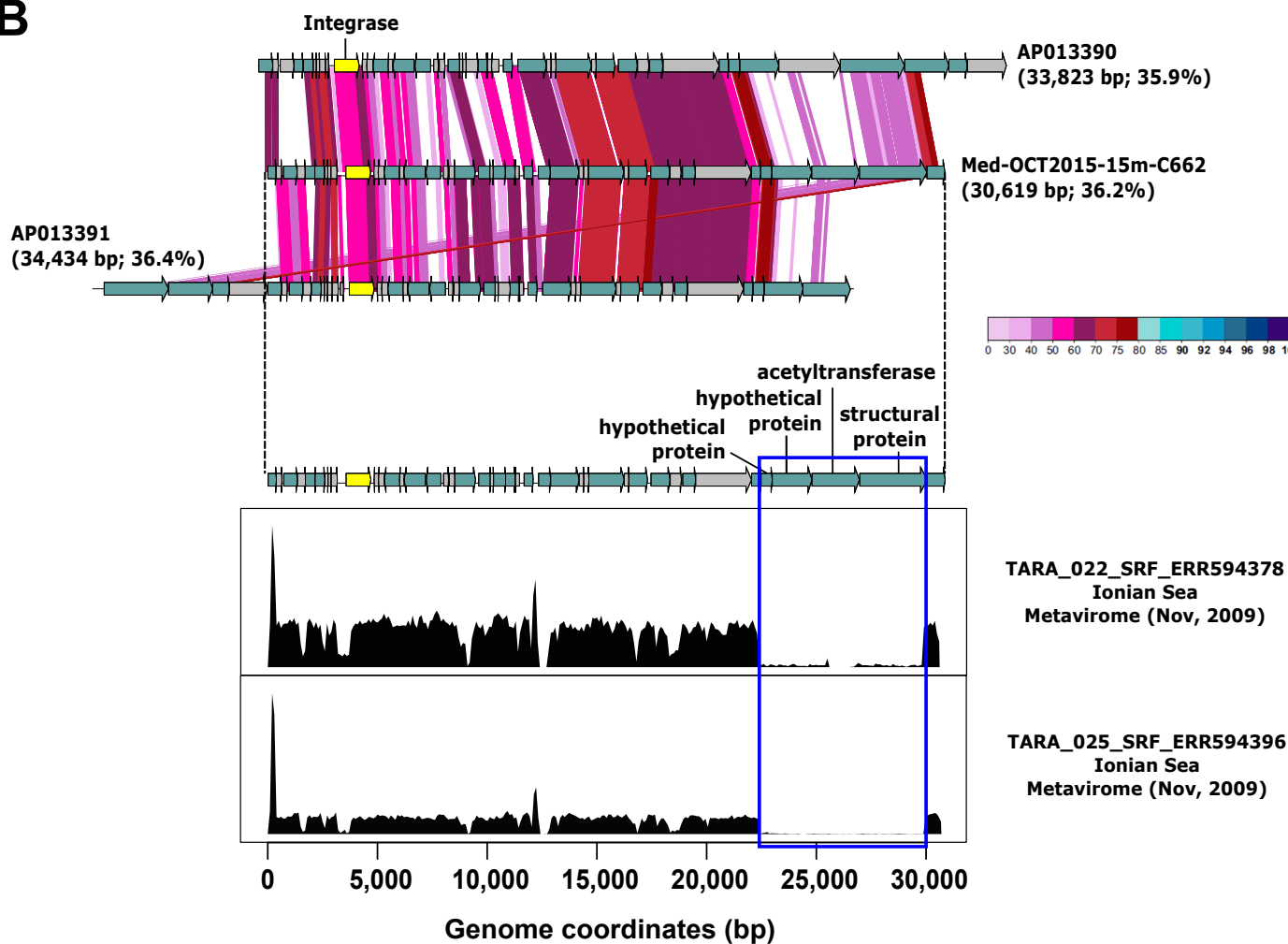

Supplement: S8 Fig — (A) Two CGRs in comparison to the cultivated pelagiphage HTVC019P and the uvMED genome AP013545. (B). Genome comparison between Med-OCT2015-15m-C662 and members of the previously described G15 group related to pelagiphage HTVC010P. (PDF) [file pgen.1007018.s008.pdf]

**A**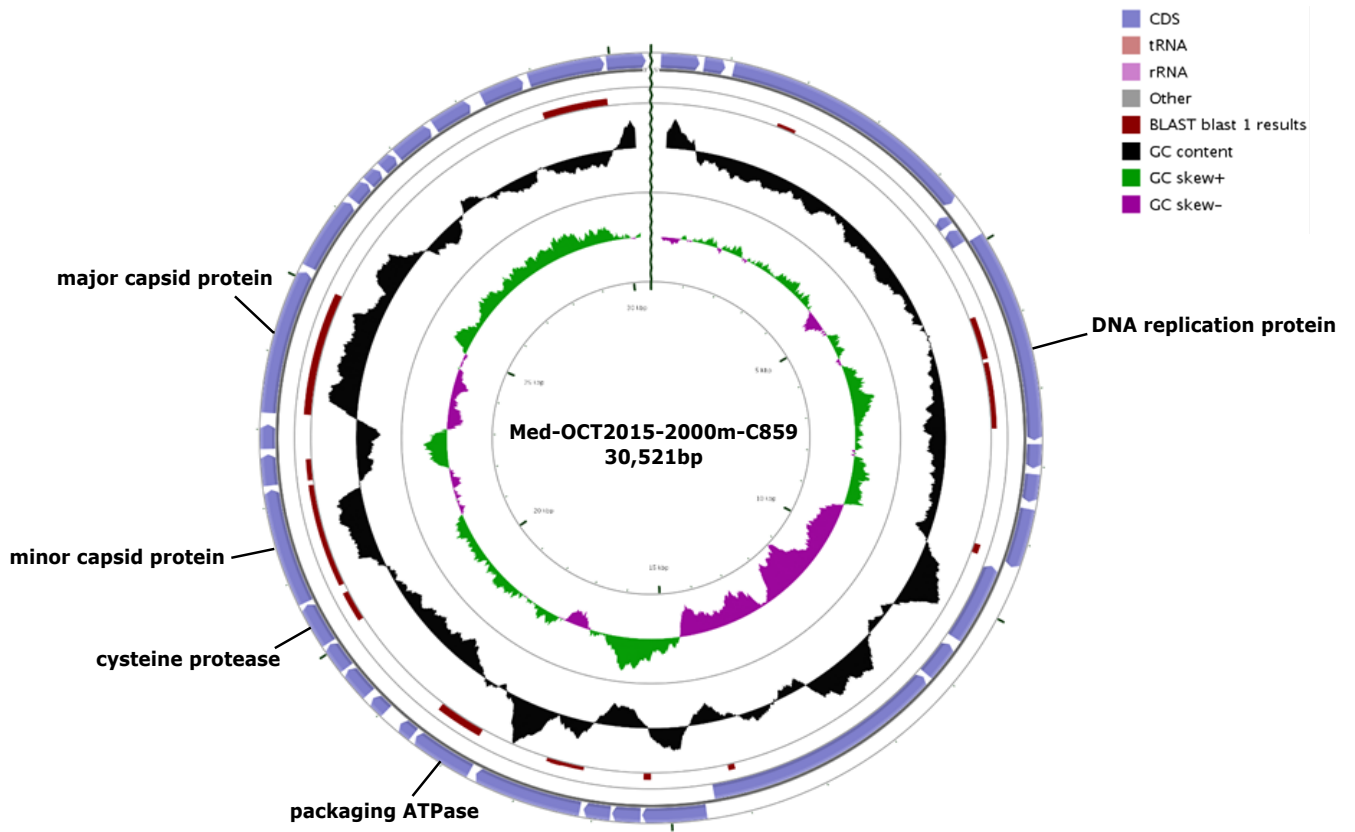**B**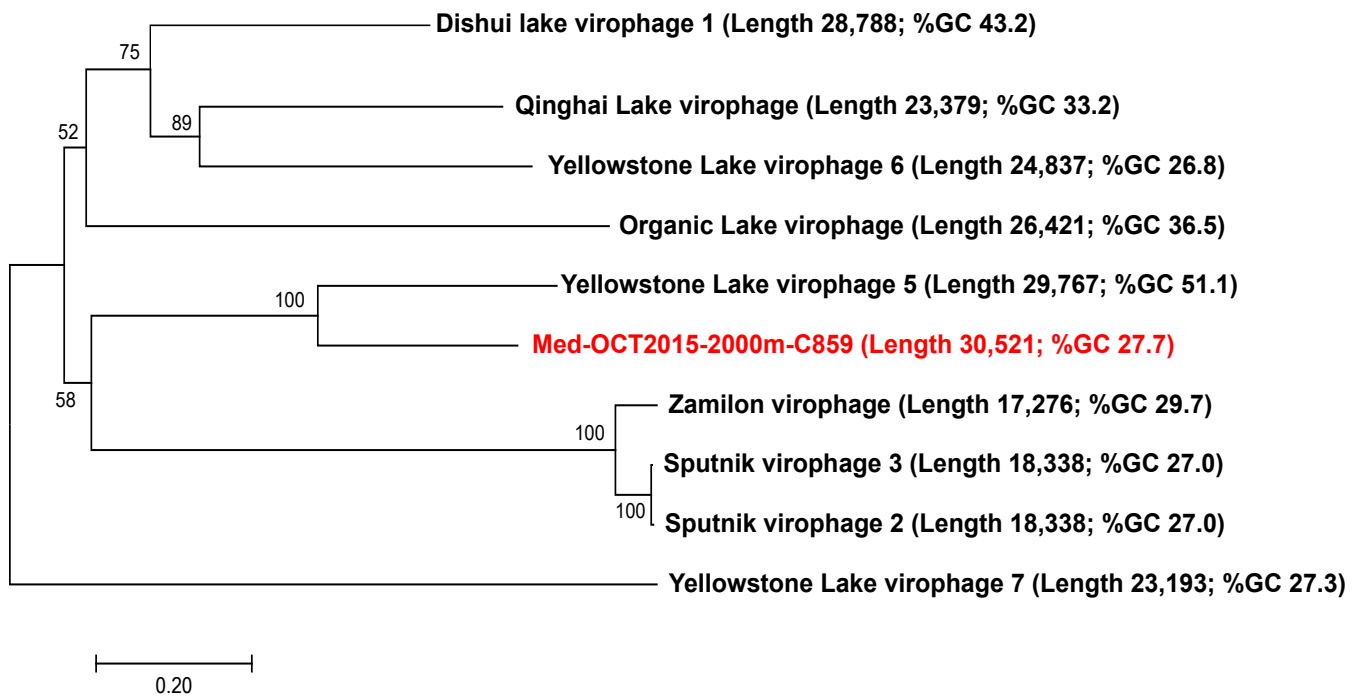

Supplement: S10 Fig — (A) Complete genome of Med-OCT2015-2000m-C859, homologous genes in other virophages are labeled. (B) Maximum-likelihood-based phylogenetic analysis of the concatenation of seven shared amino acid sequences with other already described virophages. (PDF) [file pgen.1007018.s010.pdf]

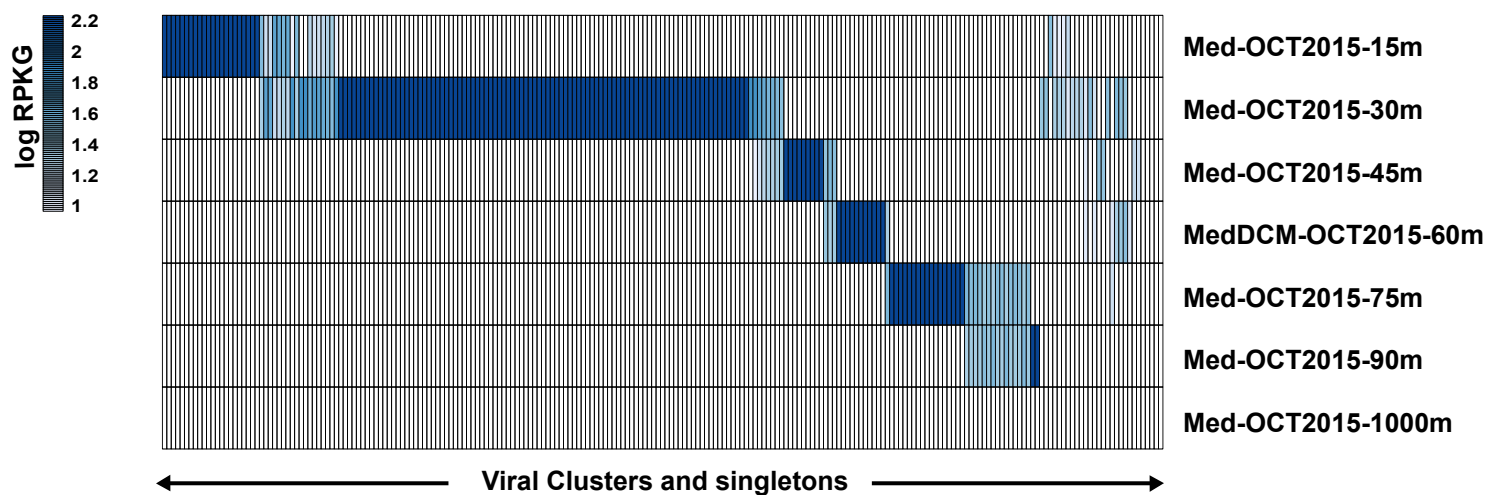

Supplement: S11 Fig — We took into consideration only those Viral clusters recruiting more than 10 RPKG. (PDF) [file pgen.1007018.s011.pdf]

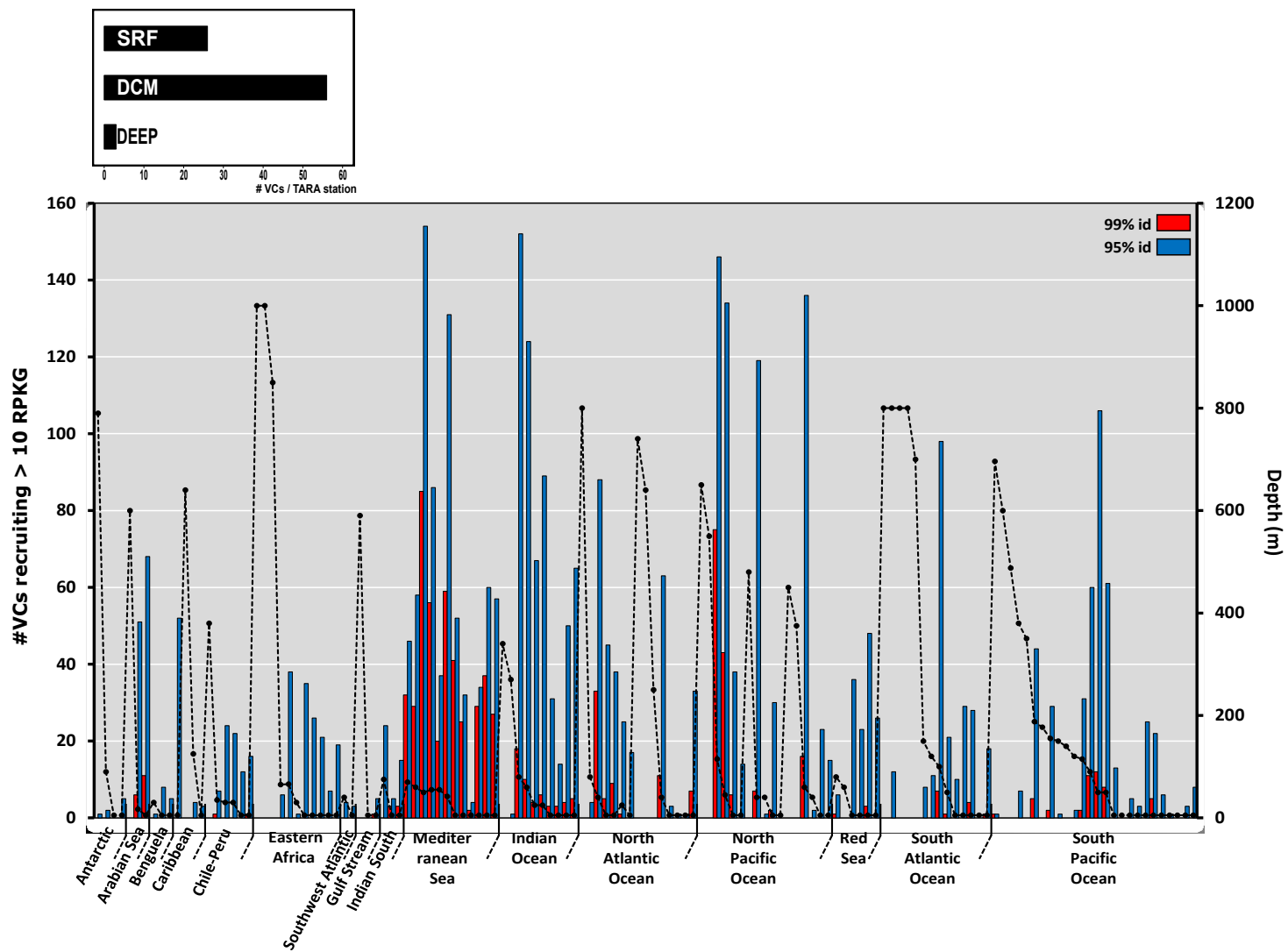

Supplement: S12 Fig — Left axis indicates depth of sample. (PDF) [file pgen.1007018.s012.pdf]
