# Supplementary material for: Genome diversity of marine phages recovered from Mediterranean metagenomes: Size matters
Source: PLoS Genet. 2017 Sep 25;13(9):e1007018. doi: 10.1371/journal.pgen.1007018 (PMC5628999; doi:10.1371/journal.pgen.1007018)
Supplement: S6 Fig — Samples highlighted in blue and red correspond to samples obtained from the 5–20μm and <0.22μm filters, respectively. (PDF) [file pgen.1007018.s006.pdf]

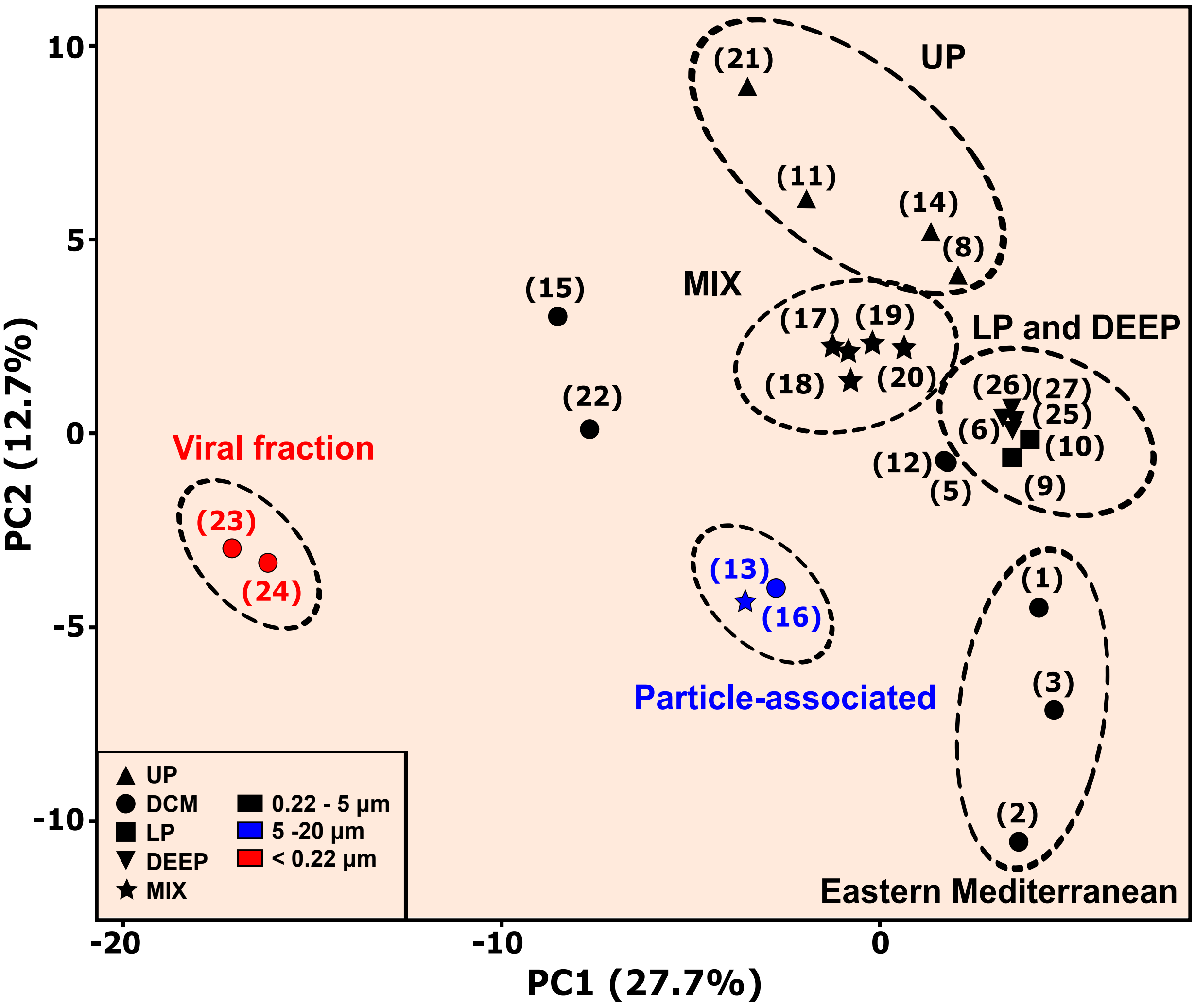

(1) Med-Ae1-75mDCM
(2) Med-Io16-70mDCM
(3) Med-Io7-77mDCM
(4) MedDCM-SEP2015_HS
(5) MedDCM-JUL2012
(6) Med-OCT2015-1000m
(7) Med-OCT2015-45m
(8) Med-OCT2015-15m
(9) Med-OCT2015-75m
(10) Med-OCT2015-90m
(11) Med-OCT2015-30m
(12) MedDCM-OCT2015-60m
(13) MedDCM-SEP2013-LF
(14) Med-SEP2014-15m
(15) MedDCM-SEP2014
(16) MedWinter-JAN2015-20m-LF
(17) MedWinter-JAN2015-80m
(18) MedWinter-JAN2015-20m
(19) MedWinter-JAN2015-Coastal
(20) MedWinter-DEC2013-20m
(21) Med-SEP2014-30m
(22) MedDCM-SEP2013
(23) MedDCM-SEP2013-Vir
(24) MedDCM-Vir-MDA
(25) Med-Ae2-600mDeep
(26) Med-OCT2015-2000m
(27) Med-Io17-3500mDeep
