# Supplementary material for: Genome diversity of marine phages recovered from Mediterranean metagenomes: Size matters
Source: PLoS Genet. 2017 Sep 25;13(9):e1007018. doi: 10.1371/journal.pgen.1007018 (PMC5628999; doi:10.1371/journal.pgen.1007018)
Supplement: S9 Fig — (PDF) [file pgen.1007018.s009.pdf]

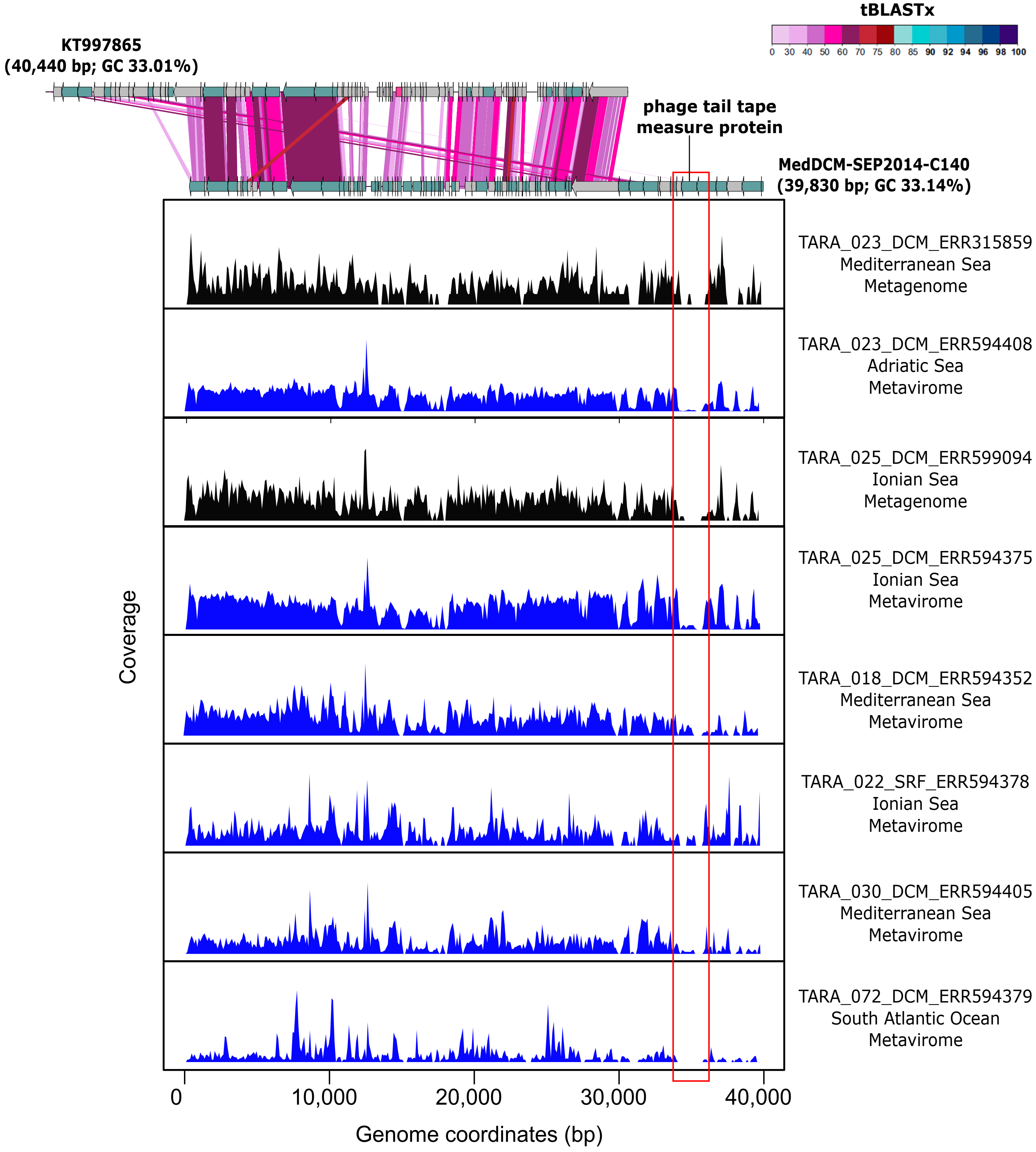
tBLASTx
0 30 40 50 60 70 75 80 85 90 92 94 96 98 100
KT997865
(40,440 bp; GC 33.01%)
phage tail tape measure protein
MedDCM-SEP2014-C140
(39,830 bp; GC 33.14%)
TARA_023_DCM_ERR315859
Mediterranean Sea
Metagenome
TARA_023_DCM_ERR594408
Adriatic Sea
Metavirome
TARA_025_DCM_ERR599094
Ionian Sea
Metagenome
TARA_025_DCM_ERR594375
Ionian Sea
Metavirome
TARA_018_DCM_ERR594352
Mediterranean Sea
Metavirome
TARA_022_SRF_ERR594378
Ionian Sea
Metavirome
TARA_030_DCM_ERR594405
Mediterranean Sea
Metavirome
TARA_072_DCM_ERR594379
South Atlantic Ocean
Metavirome
Coverage
0
10,000
20,000
30,000
40,000
Genome coordinates (bp)
